# SARS-CoV-2 incidence, transmission, and reinfection in a rural and an urban setting: results of the PHIRST-C cohort study, South Africa, 2020–21

**DOI:** 10.1016/S1473-3099(22)00069-X

**Published:** 2022-06

**Authors:** Cheryl Cohen, Jackie Kleynhans, Anne von Gottberg, Meredith L McMorrow, Nicole Wolter, Jinal N Bhiman, Jocelyn Moyes, Mignon du Plessis, Maimuna Carrim, Amelia Buys, Neil A Martinson, Kathleen Kahn, Stephen Tollman, Limakatso Lebina, Floidy Wafawanaka, Jacques D du Toit, Francesc Xavier Gómez-Olivé, Fatimah S Dawood, Thulisa Mkhencele, Kaiyuan Sun, Cécile Viboud, Stefano Tempia, PHIRST-C Group, Jinal N Bhiman, Amelia Buys, Maimuna Carrim, Cheryl Cohen, Linda de Gouveia, Mignon du Plessis, Jacques du Toit, Francesc X Gómez-Olivé, Kathleen Kahn, Kgaugelo P Kgasago, Jackie Kleynhans, Retshidisitswe Kotane, Limakatso Lebina, Neil A. Martinson, Meredith L McMorrow, Tumelo Moloantoa, Jocelyn Moyes, Stefano Tempia, Stephen Tollman, Anne von Gottberg, Floidy Wafawanaka, Nicole Wolter

**Affiliations:** a Centre for Respiratory Diseases and Meningitis, National Institute for Communicable Diseases of the National Health Laboratory Service, Johannesburg, South Africa; b School of Public Health, Faculty of Health Sciences, University of the Witwatersrand, Johannesburg, South Africa; c School of Pathology, Faculty of Health Sciences, University of the Witwatersrand, Johannesburg, South Africa; d MRC/Wits Rural Public Health and Health Transitions Research Unit (Agincourt), Faculty of Health Sciences, University of the Witwatersrand, Johannesburg, South Africa; e Influenza Division, Centers for Disease Control and Prevention, Atlanta, GA, USA; f Perinatal HIV Research Unit, University of the Witwatersrand, Johannesburg, South Africa; g Johns Hopkins University Center for TB Research, Baltimore, MD, USA; h Africa Health Research Institute, KwaZulu-Natal, South Africa; i Division of International Epidemiology and Population Studies, Fogarty International Center, National Institutes of Health, Bethesda, MD, USA

## Abstract

**Background:**

By August, 2021, South Africa had been affected by three waves of SARS-CoV-2; the second associated with the beta variant and the third with the delta variant. Data on SARS-CoV-2 burden, transmission, and asymptomatic infections from Africa are scarce. We aimed to evaluate SARS-CoV-2 burden and transmission in one rural and one urban community in South Africa.

**Methods:**

We conducted a prospective cohort study of households in Agincourt, Mpumalanga province (rural site) and Klerksdorp, North West province (urban site) from July, 2020 to August, 2021. We randomly selected households for the rural site from a health and sociodemographic surveillance system and for the urban site using GPS coordinates. Households with more than two members and where at least 75% of members consented to participate were eligible. Midturbinate nasal swabs were collected twice a week from household members irrespective of symptoms and tested for SARS-CoV-2 using real-time RT-PCR (RT-rtPCR). Serum was collected every 2 months and tested for anti-SARS-CoV-2 antibodies. Main outcomes were the cumulative incidence of SARS-CoV-2 infection, frequency of reinfection, symptomatic fraction (percent of infected individuals with ≥1 symptom), the duration of viral RNA shedding (number of days of SARS-CoV-2 RT-rtPCR positivity), and the household cumulative infection risk (HCIR; number of infected household contacts divided by the number of susceptible household members).

**Findings:**

222 households (114 at the rural site and 108 at the urban site), and 1200 household members (643 at the rural site and 557 at the urban site) were included in the analysis. For 115 759 nasal specimens from 1200 household members (follow-up 92·5%), 1976 (1·7%) were SARS-CoV-2-positive on RT-rtPCR. By RT-rtPCR and serology combined, 749 of 1200 individuals (62·4% [95% CI 58·1–66·4]) had at least one SARS-CoV-2 infection episode, and 87 of 749 (11·6% [9·4–14·2]) were reinfected. The mean infection episode duration was 11·6 days (SD 9·0; range 4–137). Of 662 RT-rtPCR-confirmed episodes (>14 days after the start of follow-up) with available data, 97 (14·7% [11·9–17·9]) were symptomatic with at least one symptom (in individuals aged <19 years, 28 [7·5%] of 373 episodes symptomatic; in individuals aged ≥19 years, 69 [23·9%] of 289 episodes symptomatic). Among 222 households, 200 (90·1% [85·3–93·7]) had at least one SARS-CoV-2-positive individual on RT-rtPCR or serology. HCIR overall was 23·9% (195 of 817 susceptible household members infected [95% CI 19·8–28·4]). HCIR was 23·3% (20 of 86) for symptomatic index cases and 23·9% (175 of 731) for asymptomatic index cases (univariate odds ratio [OR] 1·0 [95% CI 0·5–2·0]). On multivariable analysis, accounting for age and sex, low minimum cycle threshold value (≤30 *vs* >30) of the index case (OR 5·3 [2·3–12·4]) and beta and delta variant infection (*vs* Wuhan-Hu-1, OR 3·3 [1·4–8·2] and 10·4 [4·1–26·7], respectively) were associated with increased HCIR. People living with HIV who were not virally supressed (≥400 viral load copies per mL) were more likely to develop symptomatic illness when infected with SAR-CoV-2 (OR 3·3 [1·3–8·4]), and shed SARS-CoV-2 for longer (hazard ratio 0·4 [95% CI 0·3–0·6]) compared with HIV-uninfected individuals.

**Interpretation:**

In this study, 565 (85·3%) SARS-CoV-2 infections were asymptomatic and index case symptom status did not affect HCIR, suggesting a limited role for control measures targeting symptomatic individuals. Increased household transmission of beta and delta variants was likely to have contributed to successive waves of SARS-CoV-2 infection, with more than 60% of individuals infected by the end of follow-up.

**Funding:**

US CDC, South Africa National Institute for Communicable Diseases, and Wellcome Trust.

## Introduction

Large numbers of hospitalisations and deaths related to COVID-19 have occurred in many low-income and middle-income countries. However, reported rates of illness are not always proportional to the high rates of SARS-CoV-2 infection implied by serological studies, suggesting low access to laboratory testing, or differences in transmission or susceptibility to developing SARS-CoV-2-related illness in these settings.^1^, ^2^ Few detailed SARS-CoV-2 cohort studies are available from middle-income and low-income settings, where vaccination rates remain suboptimal and immunity comes primarily from natural infections, as is the case in South Africa. South Africa has a young population with less than 5% of the population older than 65 years.^3^ South Africa also has a high population HIV prevalence, which could affect SARS-CoV-2 burden and transmission if people living with HIV have increased susceptibility to infection or are more likely to transmit SARS-CoV-2. In 2017, the national HIV prevalence among individuals of all ages was 14%, with 7·9 million people living with HIV.^4^

**Research in context**

**Evidence before this study**

We searched the PubMed database from Jan 1, 2015, to Nov 31, 2021, for research papers, systematic reviews, and meta-analyses with the search terms “SARS-CoV-2” OR “COVID-19” AND “transmission” OR “household transmission” OR “symptomatic fraction”, without language restrictions. A 2020 systematic review found that 20% (95% CI 3–67) of SARS-CoV-2 infections remained asymptomatic throughout infection and that transmission from asymptomatic individuals was lower than from symptomatic individuals. A systematic review and meta-analysis of 87 household transmission studies of SARS-CoV-2 found an estimated secondary attack rate from index cases to household members of 18·9% (95% CI 16·2–22·0). The review also found that household secondary attack rates were increased from symptomatic index cases and that adults were more likely than children to acquire infection. As of December, 2021, South Africa has had three waves of SARS-CoV-2 infections; the second and third waves were associated with circulation of the beta and delta variants, respectively. Studies to quantify the burden of asymptomatic infections, symptomatic fraction, reinfection frequency, duration of SARS-CoV-2 viral shedding, and household transmission of SARS-CoV-2 from asymptomatically infected individuals have mostly been conducted as part of outbreak investigations or in specific settings. Comprehensive systematic community studies of SARS-CoV-2 burden and transmission including for the beta and delta variants are scarce, especially in settings with low vaccination coverage.

**Added value of this study**

We found a high attack rate of SARS-CoV-2, with 749 of 1200 individuals (62·4% [95% CI 58·1–66·4]) having at least one episode of SARS-CoV-2 infection on real-time RT-PCR or serology (or both RT-PCR and serology) over 13 months of follow-up (ending August, 2021), and 87 of 749 (11·6% [9·4–14·2]) having a repeat infection. 97 of 662 episodes (14·7% [11·9–17·9]) in eligible individuals (>14 days after the start of follow-up) were symptomatic (≥1 symptom); 28 of 373 episodes (7·5% [4·7–11·6]) were symptomatic in individuals younger than 19 years, and 69 of 289 episodes (23·9% [19·2–29·3]) were symptomatic in individuals aged 19 years or older. SARS-CoV-2 infected index cases transmitted the infection to 195 of 817 (23·9% [95% CI 19·8–28·4]) susceptible household contacts, similar to previous studies. Presence of symptoms in the index case was not associated with household transmission, and the odds of transmission from index cases were 3-times greater with beta variant infection and 10-times greater with delta variant infection than with Wuhan-Hu-1 infection. Attack rates were highest in individuals aged 13–18 years and individuals in this age group were more likely to experience repeat infections and to acquire SARS-CoV-2 infection within households than other age groups. People living with HIV who were not virally supressed (≥400 viral load copies per mL) were more likely to develop symptomatic illness when infected with SARS-CoV-2, and shed SARS-CoV-2 for longer, when compared to HIV-uninfected individuals. Vaccination uptake was low in the rural site and urban site, with 57 (4·8%) of 1200 individuals fully vaccinated at the end of follow up.

**Implications of all the available evidence**

We found a high rate of SARS-CoV-2 infection in households in a rural community and an urban community in South Africa, with most infections being asymptomatic in individuals of all ages. Asymptomatic individuals transmitted SARS-CoV-2 at similar levels to symptomatic individuals, suggesting that interventions targeting symptomatic individuals such as symptom-based testing and contact tracing of individuals tested because they report symptoms might have limited effects as control measures. Increased household transmission of beta and delta variants was likely to have contributed to recurrent waves of SARS-CoV-2 infection, with more than 60% of individuals infected by the end of follow-up. Increased attack rates, reinfection, and acquisition in adolescents and prolonged SARS-CoV-2 shedding in people living with HIV who were not virally suppressed suggests that prioritised vaccination of individuals in these groups could affect community transmission.

After the initial detection of SARS-CoV-2 in South Africa in March, 2020, a strict lockdown was implemented on March 27, 2020, with restrictions on international travel, school closures, halting of non-essential business, and confinement of people to their homes. Subsequent to this lockdown was a progressive relaxation of restrictions, from May 1, 2020. South Africa was affected by three SARS-CoV-2 waves up to August, 2021; the first wave due to the Wuhan-Hu-1 strain peaked in August, 2020, the second wave, associated with the emergence of the beta variant, peaked in January, 2021, and the third wave, associated with the delta variant, peaked in June, 2021.^5^ SARS-CoV-2 restrictions were increased moderately, including school closures around the peak of the second and third waves, and subsequently relaxed when case numbers decreased. Both the beta and delta variants have been shown to escape immunity from previous infection and to be more transmissible than the Wuhan-Hu-1 strain, and are possibly associated with increased severity of COVID-19, although the epidemiological consequences of each of these parameters are debated.^6^, ^7^

Studies to quantify the burden of asymptomatic infections, symptomatic fraction, reinfection frequency, duration of shedding, and household transmission of SARS-CoV-2 from asymptomatically infected individuals have mostly been conducted as part of outbreak investigations or in specific settings. Comprehensive systematic community studies of SARS-CoV-2 burden and transmission including for the beta and delta variants are scarce, especially in settings with low vaccination coverage. In the present study, in randomly selected households from a rural and an urban community in South Africa, we estimated the cumulative incidence of SARS-CoV-2 infection, the frequency of reinfection, and the correlation between infections diagnosed by serial real-time RT-PCR (RT-rtPCR) and serology. We also estimated the symptomatic fraction of SARS-CoV-2 infection, the duration of viral RNA shedding, and the household cumulative infection risk (HCIR) from symptomatic and asymptomatic index cases of different ages.

## Methods

### Study design and participants

The prospective household cohort study of SARS-CoV-2, influenza, and respiratory syncytial virus community burden, transmission dynamics, and viral interaction in South Africa (PHIRST-C) was based on a previously conducted study (PHIRST) at the same sites in 2016–18.^8^, ^9^ The rural site, Agincourt study area in Bushbuckridge subdistrict (Mpumalanga province), is within a health and sociodemographic surveillance system (HDSS) run by the Medical Research Council and University of the Witwatersrand Rural Public Health and Health Transitions Research Unit, Agincourt.^10^, ^11^ The urban site, Jouberton township in Klerksdorp, is located in the North West province (appendix pp 1–2, 25). The study included 58 weeks of follow-up at the rural site (July 16, 2020 to Aug 28, 2021) and 56 weeks of follow-up at the urban site (July 27, 2020, to Aug 28, 2021).

Households were randomly selected, from the HDSS database for the rural site and with use of GPS coordinates for the urban site (appendix p 1). Households with more than two members and where at least 75% of members consented to participate were eligible. In brief, we first approached households previously enrolled in PHIRST, and then prospectively approached new potentially eligible households using the site-specific sampling frame used for PHIRST until the required number of households were enrolled (appendix pp 1–2).

The PHIRST protocol was approved by the University of the Witwatersrand (Johannesburg, South Africa) Human Research Ethics Committee (reference 150808) and an amendment to include enrolment and testing for COVID-19 was approved by the same committee on June 24, 2020. The protocol was registered on ClinicalTrials.gov on Aug 6, 2015, and updated on Dec 30, 2020 and March 10, 2022 (NCT02519803). We obtained informed consent from all adult participants (aged ≥18 years), assent from children aged 7 to 17 years, and consent from a parent or guardian for children younger than 18 years before data collection. Participants received grocery store vouchers of US$3 per visit to compensate for time required for specimen collection and interview.

### Data collection

We collected individual baseline data, including demographics, HIV status, and clinically diagnosed underlying illness. All baseline data were self-reported apart from HIV status, which was confirmed from medical records or testing (appendix p 5). Midturbinate nasal swabs, COVID-19 related symptoms, and health-seeking data were collected or recorded twice a week, and serum was collected every 2 months to measure SARS-CoV-2 antibodies, with a total of seven serum collections at each site. Nasal swab collection began before the first SARS-CoV-2 wave peak in the Mpumalanga province where the rural site is located, and during the first wave peak in North West province where the urban site is located.^12^ Study staff visited participating households twice a week (Monday–Wednesday and Thursday–Saturday) from July, 2020 to August, 2021, to collect the midturbinate nasal swabs and information about symptoms, absenteeism, and health system contact. Different COVID-19-related symptoms were captured for young children (aged <5 years) compared with older children and adults (≥5 years; appendix pp 4–5, 12). Data were entered during visits on tablet computers with use of the Research Electronic Data Capture application.^13^ Blood specimens were collected at enrolment (from July 20 to Sept 17, 2020, blood draw 1), and every 2 months thereafter (Sept 21 to Oct 10, 2020, blood draw 2; Nov 23 to Dec 12, 2020, blood draw 3; Jan 25 to Feb 21, 2021, blood draw 4; March 22 to April 11, 2021, blood draw 5; May 20 to June 9, 2021, blood draw 6; and July 19 to Aug 5, 2021, blood draw 7). Serology data from the first five blood draws have been published.^2^

### Laboratory methods

Nasal specimens were collected with nylon flocked swabs, placed in universal transport medium (UTM), and transported twice a week on ice packs to the National Institute for Communicable Diseases in Johannesburg, South Africa, for testing. All laboratory kits were used according to manufacturer instructions. Nucleic acids were extracted from 200 μl universal transport medium with the Microlab NIMBUS instrument (Hamilton, NV, USA) and the STARMag Universal Cartridge extraction kit (Seegene; Seoul, South Korea). Specimens were tested for the presence of SARS-CoV-2 nucleic acids by RT-rtPCR initially with the Allplex 2019-nCoV Assay (Seegene; Seoul, South Korea) and a Bio-Rad CFX96 thermal cycler (Bio-Rad Laboratories; Hercules, CA, USA). From March 1, 2021, samples were tested with the Allplex SARS-CoV-2/FluA/FluB/RSV Assay (Seegene; Seoul, South Korea). A cycle threshold (Ct) value below 40 on at least one of three SARS-CoV-2 PCR targets (E or S gene and N and RdRp genes), confirmed on repeat RT-rtPCR testing, was considered positive. On repeat testing, extraction was performed on a single aliquot; PCR was run with each extract loaded in duplicate. A low Ct value on RT-rtPCR (based on the lowest Ct value for any target during the SARS-CoV-2 episode) was used as a proxy for high RNA viral load.^14^ All confirmed positive samples were tested with Seegene Allplex Variants I and Variants II typing assays (Seegene; Seoul, South Korea) which differentiate variants alpha (B.1.1.7), beta (B.1.351), delta (B1.617.2), and gamma (P.1). Confirmed positive samples were sequenced if RT-rtPCR Ct values were 35 or lower (appendix p 6).

Serological evidence of SARS-CoV-2 infection was established with the Roche Elecsys Anti-SARS-CoV-2 assay (Roche Diagnostics; Rotkreuz, Switzerland), with use of recombinant protein representing the nucleocapsid antigen. The assay was performed on the Cobas e601 instrument (Roche Diagnostics; Rotkreuz, Switzerland), and a cutoff index of 1·0 or higher was considered an indication of infection (seropositivity).

Vaccination status was assessed at each SARS-CoV-2 serum collection visit once vaccines became available. Individuals were considered fully vaccinated for SARS-CoV-2 when at least 14 days after receipt of a single dose of the Johnson & Johnson (Ad.26.COV2.S) vaccine or two doses of the Pfizer BioNTech (BNT162b2) vaccine and partially vaccinated if they had received any vaccine dose but did not meet the full criteria.

### Outcomes

The prespecified primary objectives were to estimate the community burden of SARS-CoV-2 including the cumulative incidence, proportion of individuals reinfected, symptomatic fraction, and fraction seeking medical care (clinic attendance and hospitalisation), and to assess the transmission dynamics, including estimation of HCIR, generation interval, and duration of viral RNA shedding. Secondary prespecified objectives included estimation of the correlation between infections diagnosed by serial RT-rtPCR and serology; estimation of the community burden and transmission dynamics by age group, HIV status, and other probable biologically associated factors with available data; and the assessment of SARS-CoV-2 transmission from asymptomatic and symptomatic individuals. Other prespecified objectives outlined in the protocol are not reported in this paper. In a post-hoc analysis, we estimated the infection fatality ratio (IFR) by dividing the number of individuals who died during an infection episode by the number of infection episodes included in analysis of symptoms and outcomes.

### Definitions and statistical analysis

We aimed to enrol 1000 individuals of all ages. Assuming an average household size of five individuals and loss to follow up of 10%, we planned to enrol approximately 110 households from each site (appendix p 2).

We excluded individuals with fewer than 11 completed follow-up visits from our analyses. We defined individuals with serology-confirmed SARS-CoV-2 infection as those with at least one instance of SARS-CoV-2 antibody seropositivity. We defined an episode of RT-rtPCR-confirmed SARS-CoV-2 infection when at least one nasal swab tested positive for SARS-CoV-2. Infection episode duration as a proxy for duration of viral shedding was estimated from the first day of RT-rtPCR positivity to the first day a test was negative. Additional positive samples testing RT-rtPCR positive within 4 weeks of the last RT-rtPCR were considered part of the same episode (appendix p 26). A COVID-related symptomatic illness episode was defined when at least one symptom was reported in the period extending from one visit before to one visit after the SARS-CoV-2 infection episode. People living with HIV were deemed immunocompromised if their CD4 T-lymphocyte count was lower than 200 cells per μl, and not HIV virally suppressed if their HIV viral load was 400 copies per mL or higher.^15^

An RT-rtPCR-confirmed household cluster was composed of all RT-rtPCR-confirmed infection episodes in a household within an interval of 14 days or less (representing ≤2 mean serial intervals) between the RT-rtPCR-positive tests of any infection episodes. Clusters could be comprised of an index case with no secondary cases.^16^ Cluster duration was estimated as the interval from the first day of RT-rtPCR positivity of the first individual in a cluster to the last day of RT-rtPCR positivity of the last individual in that cluster. HCIR was defined as the proportion of household members with subsequent infection following SARS-CoV-2 introduction and estimated by dividing the number of subsequent individuals with RT-rtPCR-confirmed infection within a household cluster following SARS-CoV-2 introduction by the number of susceptible (no evidence of previous infection on RT-rtPCR or serology) household members. The index case was defined as the first individual testing positive on RT-rtPCR within a cluster. The generation interval was calculated as the time between the date of the first positive RT-rtPCR test for the index case and the date of the first positive RT-rtPCR test for secondary infection within a household cluster.

A variant was allocated to each episode of infection according to a hierarchical process: first, if at least one sample within an identified episode had a confirmed lineage (ie, Wuhan-Hu-1, alpha, beta, or delta variant), then the lineage was assigned to the entire episode; second, if no samples within an episode had a confirmed lineage, but the episode was within a household cluster with at least one episode with a confirmed lineage, then the cluster lineage was assigned to the episode; and finally, if neither of the previous conditions were met, then the lineage was assigned on the basis of the SARS-CoV-2 wave (wave 1, 2, or 3) as a proxy for lineage circulation (appendix pp 7–8). Variants of concern were classified according to WHO definitions.

We defined possible SARS-CoV-2 reinfection when there was an interval of 28 days to 90 days inclusive between RT-rtPCR-positive specimens (no sequence or variant data available) or between the first seropositive specimen and an RT-rtPCR-positive specimen; probable reinfection when the interval was more than 90 days between RT-rtPCR-positive specimens (no sequence or variant data available) or between the first seropositive specimen and RT-rtPCR-positive specimen; and confirmed reinfection as distinct Nextstrain clades on sequencing or variant PCR between RT-rtPCR-positive specimens meeting the temporal criteria for possible or probable.^17^ The proportion of reinfections was calculated as the number of individuals with reinfection divided by the total number of individuals with evidence of previous infection.

For analyses of symptomatic fraction, infection episode duration, HCIR, and generation interval, we only included incident episodes with onset more than 14 days after the start of follow-up. This sampling was to account for some individuals who tested positive at the start of follow-up (n=7 at the rural site and n=32 at the urban site), as we did not know how long they had been shedding SARS-CoV-2, if they had symptoms previously, or who the index case was. Further definitions of the analysis populations are provided in the appendix (pp 7–9).

Proportions were compared with χ^2^ test or Fisher's exact test. We obtained 95% confidence intervals for estimated proportion accounting for household and site clustering using the svy command in Stata (version 14.1). We used Weibull accelerated failure time regression for the analysis of factors associated with time-to-event outcomes (duration of shedding and generation interval). We used logistic regression for the analysis of variables associated with binary outcomes (symptomatic *vs* asymptomatic, HCIR *vs* no risk, index case *vs* other household members, reinfection *vs* no reinfection). We used Poisson regression for the analysis of variables associated with at least one SARS-CoV-2 infection episode (cumulative incidence) including all individuals with evidence of infection on RT-rtPCR or serology. For all analyses we accounted for within-household clustering using random-effects regression models. For each univariate analysis, we used all available case information. For each multivariable model, we considered a priori all probable biologically associated factors with the outcome of interest for which we had available data. Pairwise interactions were assessed graphically and by inclusion of product terms for all variables remaining in the final multivariable additive model. We conducted all statistical analyses using Stata (version 14.1). p values less than 0·05 were considered to indicate statistical significance or significance was inferred from 95% CIs.

### Role of the funding source

The sponsors of the study had no role in study design, data collection, data analysis, data interpretation, or writing of the report.

## Results

We approached 537 households, of which 236 (43·9%) had more than two household members, and the head of household agreed to participate in the study. Of these households, 222 (94·1%; 114 at the rural site and 108 at the urban site) met all inclusion criteria and were included in the analysis (appendix p 27). Of 1251 eligible household members in 222 households, 1200 (95·9%; 643 in the rural community and 557 in the urban community) were included in the analysis (appendix p 27). Among the 222 included households, the median number of household members was five (IQR 4–7), median sleeping rooms was three (IQR 2–4), and 109 (49·1%) of 222 had at least one child younger than 5 years, with a higher proportion of households having young children in the rural community (appendix pp 13–16). Of 1200 household members, 154 (12·8%) were younger than 5 years, 340 (28·3%) were aged 5–12 years, 170 (14·2%) were aged 13–18 years, 265 (22·0%) were aged 19–39 years, 168 (14·0%) were aged 40–59 years, and 103 (8·6%) were aged 60 years or older. Individuals from the rural community were younger, had a lower level of formal education, and were less likely to be employed. Underlying illness was more common in the urban community, but HIV prevalence was similar between sites (84 [13·1%] individuals with known infection in the rural community and 92 [16·5%] in the urban community, p=0·15). At the end of follow-up, 57 (4·8%) individuals across both sites were fully vaccinated against SARS-CoV-2 (appendix pp 13–16).

**Figure 1:**
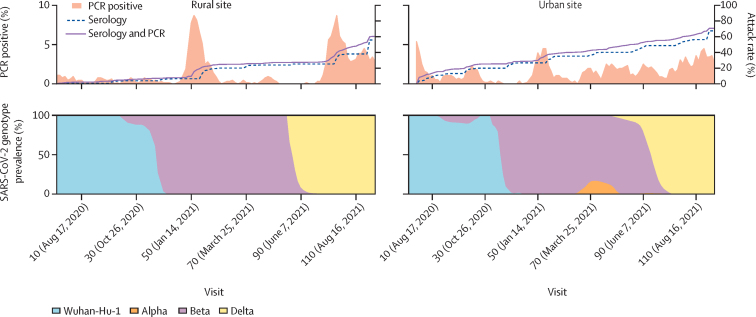
Percentage of participants with SARS-CoV-2 infection and genotype prevalence among cases over time The top panel shows the percentage of participants testing RT-rtPCR-positive for SARS-CoV-2 per study visit and the cumulative percentage with evidence of infection (attack rate) on serology only and on RT-rtPCR and serology combined. The bottom panel shows the percentage of RT-rtPCR-positive samples typed as Wuhan-Hu-1 or variants of concern (alpha, beta, or delta) by follow-up visit. A ten-visit moving average was used for smoothing in all plots. Numbering of visits accounted for the lag in initiation at the urban site (urban site started on a higher visit number). RT-rtPCR=real-time RT-PCR.

**Figure 2:**
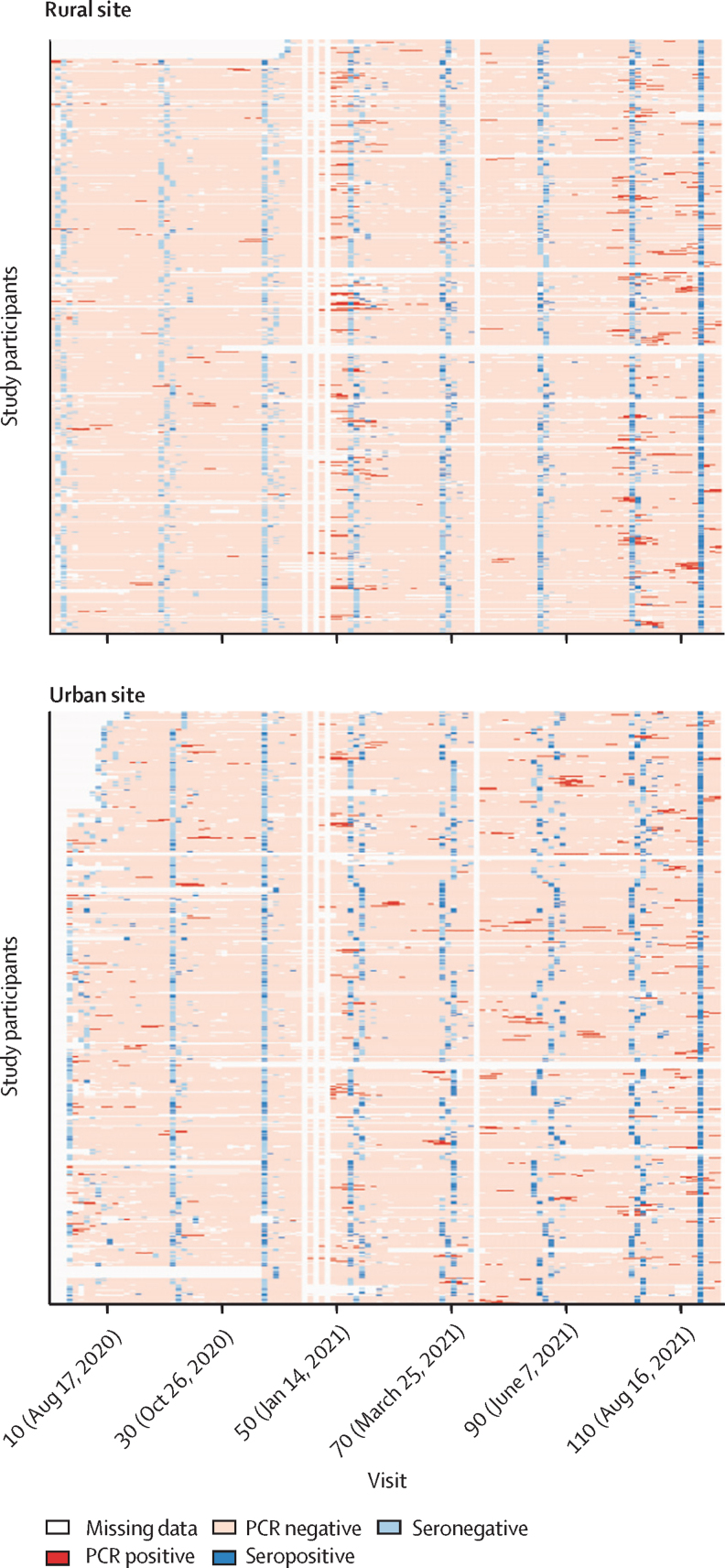
Results of serology and real-time RT-PCR among 1200 individuals (rural site, n=643; urban site, n=557) Columns are individual follow-up visits and rows are individual participants. Individuals within the same household are numbered consecutively (appear below one another). Data were missing if no sample was tested. Cells at the time of serology blood draws are coloured according to the results of serology. Numbering of visits accounted for the lag in initiation at the urban site (urban site started on a higher visit number).

At the first blood draw in July–August, 2020, among individuals with available data, five (1·1%) of 443 at the rural site and 73 (14·7%) of 498 at the urban site had serological evidence of previous SARS-CoV-2 infection (figure 1). Of 125 088 potential individual follow-up visits, we collected and tested 115 759 (92·5%) midturbinate nasal swabs, of which 1976 (1·7%) tested positive for SARS-CoV-2 on RT-rtPCR (figure 2, appendix pp 29–32). During the study, 200 of 222 households (90·1% [95% CI 85·3–93·7]) had at least one individual testing SARS-CoV-2-positive on RT-rtPCR or serology, with a mean of 3·7 (SD 2·0, range 1–10) infected individuals (irrespective of number of episodes) per infected household.

**Figure 3:**
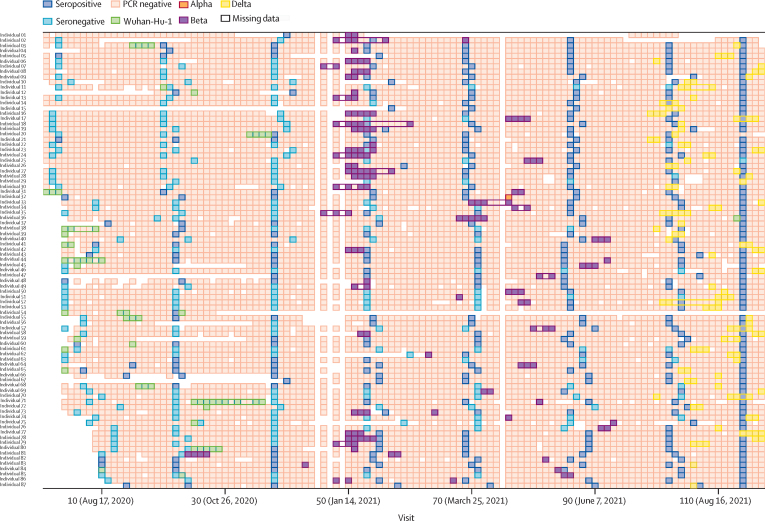
Timing of results of serology and RT-rtPCR among 87 individuals with confirmed, probable, or possible SARS-CoV-2 reinfections Columns are individual follow-up visits and rows are individual participants. Data were missing if no sample was tested. RT-rtPCR-positive follow-up visits are coloured according to infecting SARS-CoV-2 variant. Infection episodes are outlined in corresponding colours. Within an episode some visits might test negative or be missed. Cells at the time of serology blood draws are coloured according to the results of serology. RT-rtPCR=real-time RT-PCR. Numbering of visits accounted for the lag in initiation at the urban site (urban site started on a higher visit number).

During the follow-up period, 749 of 1200 individuals (62·4% [58·1–66·4]) had at least one episode of SARS-CoV-2 infection on RT-rtPCR, serology, or both RT-rtPCR and serology, and 87 of 749 (11·6% [9·4–14·2]) had a repeat infection (one individual had two repeat infections). Of 88 repeat infection episodes, 10 (11·4%) were possible reinfection, 21 (23·9%) were probable reinfection, and 57 (64·8%) were confirmed reinfection (figure 3). The highest proportion of the sample population infected was in the 13–18 years age group (appendix p 28). Repeat infection was more common in individuals aged 13–18 years (*vs* <5 years group) and in the urban community (appendix pp 17–18).

294 individuals had a positive RT-rtPCR during follow-up, negative serology preceding the episode, and available serology data more than 14 days after the start of the episode. Among these individuals, 267 (90·8%) seroconverted after the episode. Absence of a serological response was more common in children younger than 5 years and adults aged 19–59 years (*vs* 5–12 years) and for episodes with a short duration of viral shedding (≤4 days) or high minimum Ct value (>30 *vs* ≤30; appendix pp 19–20). Among 447 individuals who were seronegative at baseline and subsequently became seropositive, 404 (90·4%) had evidence of RT-rtPCR-confirmed infection during follow-up.

On multivariable analysis, factors associated with SARS-CoV-2 cumulative incidence were age 5–18 years (*vs* <5 years), living in an urban community (*vs* rural), and having a body-mass index in the overweight range (*vs* normal weight range; table 1). Of 718 RT-rtPCR-confirmed episodes, 124 (17·3%) occurred in the first wave of SARS-CoV-2 at each site, 270 (37·6%) in the second wave, and 324 (45·1%) in the third wave (appendix p 33). Proportionately more children and adolescents were infected with successive waves; in the first wave, 53 (42·7%) of 124 individuals infected were younger than 19 years, compared with 133 (49·3%) of 270 in the second wave, and 213 (65·7%) of 324 in the third wave (p<0·0001). 662 RT-rtPCR-confirmed episodes occurred more than 14 days after the start of follow-up. Among these, 97 episodes (14·7% [11·9–17·9]) were symptomatic with at least one COVID-19 related symptom (appendix p 10). In individuals younger than 19 years, 28 of 373 episodes (7·5% [4·7–11·6]) were symptomatic; in individuals aged 19 years or older, 69 of 289 episodes (23·9% [19·2–29·3]) were symptomatic. Among the 97 symptomatic episodes, six (6·2%) led to an outpatient clinic visit, nine (9·3%) to hospitalisation, and two (2·1%) resulted in death (infection fatality ratio 0·3% (two deaths among 662 infection episodes; 95% CI 0·03–1·00). Among 20 symptomatic individuals who were employed or attended school, seven (35·0%) reported absenteeism. In multivariable analysis, symptoms were more common in individuals aged 19 years or older (*vs* 5–12 years), people living with HIV with higher HIV viral load (≥400 copies per mL; *vs* uninfected HIV status), individuals with obesity (*vs* normal weight), in episodes with a low minimum Ct value (≤30), and in episodes caused by the delta variant (*vs* Wuhan-Hu-1; table 2). Positive serology before the episode was associated with reduced symptoms.

**Table 1:** Factors associated with cumulative incidence of SARS-CoV-2 infection on real-time RT-PCR or serology among 1200 individuals

	**SARS-CoV-2 infection, n/N (%)**	Univariate RR*(95% CI)	Multivariable adjusted RR*(95% CI)
**Site**			
Rural	368/643 (57·2%)	1 (ref)	1 (ref)
Urban	381/557 (68·4%)	1·9 (1·2–3·0)	1·7 (1·1–2·7)
**Age group, years**			
<5	75/154 (48·7%)	1 (ref)	1 (ref)
5–12	205/340 (60·3%)	2·0 (1·2–3·2)	1·8 (1·1–2·9)
13–18	132/170 (77·6%)	5·1 (2·8–9·2)	4·4 (2·4–8·1)
19–39	165/265 (62·3%)	1·9 (1·1–3·0)	1·5 (0·9–2·6)
40–59	115/168 (68·5%)	2·7 (1·5–4·7)	2·1 (0·9–4·2)
≥60	57/103 (55·3%)	1·5 (0·8–2·8)	1·2 (0·6–2·4)
**Sex**			
Female	454/717 (63·3%)	1·1 (0·8–1·5)	..
Male	295/483 (61·1%)	1 (ref)	..
**HIV status and viral load copies per mL**†‡			
Uninfected	608/971 (62·6%)	1 (ref)	..
Infected <400 copies	87/136 (64·0%)	1·1 (0·7–1·7)	..
Infected ≥400 copies	22/31 (71·0%)	1·5 (0·6–3·8)	..
HIV status or viral load unknown	32/62 (51·6%)	0·5 (0·3–1·1)	..
**HIV status and CD4 T-cell count per μL**†§			
Uninfected	608/971 (62·6%)	1 (ref)	..
Infected ≥200 CD4 cells	99/151 (65·6%)	1·1 (0·7–1·7)	..
Infected <200 CD4 cells	8/14 (57·1%)	1·0 (0·3–3·7)	..
HIV status or CD4 cell count unknown	34/64 (53·1%)	0·6 (0·3–1·2)	..
**Other underlying illness**¶			
Absent	672/1075 (62·5%)	1 (ref)	..
Present	77/125 (61·6%)	0·8 (0·5–1·3)	..
**BMI**‖			
Underweight	55/85 (64·7%)	1·3 (0·7–2·3)	1·1 (0·6–2·1)
Normal weight	371/642 (57·8%)	1 (ref)	1 (ref)
Overweight	150/219 (68·5%)	1·7 (1·1–2·5)	1·6 (1·1–2·6)
Obese	171/252 (67·9%)	1·5 (1·0–2·1)	1·4 (0·9–2·2)
**Number of individuals in household**			
3–5	311/511 (60·9%)	1 (ref)	..
6–10	369/571 (64·6%)	1·3 (0·8–2·1)	..
≥11	69/118 (58·5%)	1·0 (0·3–2·9)	..
**Crowding (>2 people per sleeping room)**			
No	381/640 (59·5%)	1 (ref)	..
Yes	368/560 (65·7%)	1·5 (0·9–2·4)	..

RR=relative risk. BMI=body-mass index. Additional variables evaluated but not found to be significant on univariate or multivariable analysis were use of alcohol, current or previous smoking, current or previous tuberculosis, household income, fuel used for cooking, and main water source.

* Estimated with Poisson regression adjusted for clustering by site and household.

† HIV data available for 1147 (95·6%) of 1200 individuals.

‡ Among 176 people living with HIV, 167 (94·9%) had available data on HIV viral load.

§ Among 176 people living with HIV, 165 (93·8%) had available data on CD4 T-cell count.

¶ Self-reported history of asthma, lung disease, heart disease, stroke, spinal cord injury, epilepsy, organ transplant, immunosuppressive therapy, organ transplantation, cancer, liver disease, renal disease, or diabetes.

‖ BMI missing for two individuals; BMI was calculated with the formula (weight in kg)/(height in m^2^); underweight (age <18 years) was defined as weight for age or BMI <–2 SDs of the WHO Child Growth Standards; underweight (age ≥18 years) as BMI <18·5kg/m^2^; overweight (age <18 years) as BMI >+1 and ≤+2 SDs of the WHO Child Growth Standards; overweight (age ≥18 years) as BMI ≥25 kg/m^2^ and <30 kg/m^2^; obese (age <18 years) as BMI >+2 SDs of the WHO Child Growth Standards; and obese (age ≥18 years) as BMI ≥30 kg/m^2^.

**Table 2:** Factors associated with symptomatic illness* among 662 RT-rtPCR-confirmed SARS-CoV-2 episodes

	**Symptomatic illness, n/N (%)**	**Univariate OR**†**(95% CI)**	**Multivariable adjusted OR**†**(95% CI)**
**Age group, years**			
<5	6/65 (9·2%)	1·6 (0·6–4·5)	2·0 (0·7–5·8)
5–12	11/184 (6·0%)	1 (ref)	1 (ref)
13–18	11/124 (8·9%)	1·5 (0·6–3·6)	1·9 (0·8–4·7)
19–39	26/152 (17·1%)	3·2 (1·5–6·8)	4·1 (1·9–8·9)
40–59	24/83 (28·9%)	6·4 (3·0–13·9)	6·8 (2·8–16·3)
≥60	19/54 (35·2%)	8·5 (3·7–19·5)	12·5 (4·7–33·4)
**Sex**			
Female	65/403 (16·1%)	1·3 (0·8–2·1)	..
Male	32/259 (12·4%)	1 (ref)	..
**HIV status and viral load copies per mL**‡			
Uninfected	75/535 (14·0%)	1 (ref)	1 (ref)
Infected <400 copies	9/68 (13·2%)	1·0 (0·5–2·0)	0·5 (0·2–1·1)
Infected ≥400 copies	10/25 (40·0%)	4·2 (1·8–9·6)	3·3 (1·3–8·4)
HIV status or viral load unknown	3/34 (8·8%)	0·6 (0·2–2·0)	0·5 (0·1–1·7)
**HIV status and CD4 T-cell count per μL**‡			
Uninfected	75/535 (14·0%)	1 (ref)	..
Infected ≥200 CD4 cells	16/83 (19·3%)	1·5 (0·8–2·7)	..
Infected <200 CD4 cells	3/8 (37·5%)	3·7 (0·9–16·0)	..
HIV status or CD4 cell count unknown	3/36 (8·3%)	0·6 (0·2–1·9)	..
**Other underlying illness**§			
Absent	78/602 (13·0%)	1 (ref)	..
Present	19/60 (31·7%)	3·1 (1·6–5·3)	..
**BMI**¶			
Underweight	4/53 (7·5%)	0·7 (0·3–2·2)	0·6 (0·2–2·0)
Normal weight	34/341 (10·0%)	1 (ref)	1 (ref)
Overweight	22/124 (17·7%)	1·9 (1·1–3·5)	1·1 (0·6–2·3)
Obese	37/141 (26·2%)	3·2 (1·9–5·4)	2·2 (1·2–4·1)
**Duration of viral RNA shedding, days**			
≤4	10/138 (7·2%)	1 (ref)	..
>4	87/524 (16·6%)	2·5 (1·3–5·0)	..
**Minimum cycle threshold value**			
≤30	89/547 (16·3%)	2·6 (1·2–5·5)	2·6 (1·1–5·8)
>30	8/115 (7·0%)	1 (ref)	1 (ref)
**Seropositive before the episode**‖			
No	89/552 (16·1%)	1 (ref)	1 (ref)
Yes	8/102 (7·8%)	0·4 (0·2–0·9)	0·4 (0·2–0·9)
**SARS-CoV-2 vaccine status**			
Unvaccinated	82/609 (13·5%)	1 (ref)	1 (ref)
Partially vaccinated	4/9 (44·4%)	5·1 (1·4–19·5)	0·9 (0·2–4·1)
Fully vaccinated	11/44 (25·0%)	2·1 (1·1–4·4)	0·6 (0·2–1·5)
**Epidemic COVID-19 wave**			
1	7/69 (10·1%)	1 (ref)	..
2	43/269 (16·0%)	1·7 (0·7–3·9)	..
3	47/324 (14·5%)	1·5 (0·6–3·4)	..
**Variant**			
Wuhan-Hu-1	7/67 (10·4%)	1 (ref)	1 (ref)
Beta	43/263 (16·3%)	1·7 (0·7–3·9)	1·8 (0·7–4·6)
Alpha	0/7	NE	NE
Delta	47/325 (14·5%)	1·4 (0·1–3·3)	2·6 (1·1–6·6)

The analysis was restricted to 662 episodes of RT-rtPCR-confirmed SARS-CoV-2 infection with onset more than 14 days after the start of follow-up. RT-rtPCR=real-time RT-PCR. OR=odds ratio. BMI=body-mass index. NE=not estimated.

* One or more symptom *vs* no symptom reported.

† Estimated with logistic regression adjusted for clustering by site and household.

‡ HIV data available for 629 (95·0%) of 662 individuals eligible for this analysis; among 94 people living with HIV eligible for this analysis, 93 (98·9%) had available data on HIV viral load and 91 (96·8%) had available data on CD4 T-cell count.

§ Self-reported history of asthma, lung disease, heart disease, stroke, spinal cord injury, epilepsy, organ transplant, immunosuppressive therapy, organ transplantation, cancer, liver disease, renal disease, or diabetes.

¶ BMI missing for three episodes; calculated with the formula (weight in kg)/(height in m^2^); underweight (age <18 years) was defined as weight for age or BMI <–2 SDs of the WHO Child Growth Standards; underweight (age ≥18 years) as BMI <18·5kg/m^2^; overweight (age <18 years) as BMI >+1 and ≤+2 SDs of the WHO Child Growth Standards; overweight (age ≥18 years) as BMI ≥25 kg/m^2^ and <30 kg/m^2^; obese (age <18 years) as BMI >+2 SDs of the WHO Child Growth Standards; and obese (age ≥18 years) as BMI ≥30 kg/m^2^.

‖ Data missing for eight episodes.

The mean infection episode duration was 11·6 days (SD 9·0; range 4–137) and 138 (20·8%) of 662 episodes were RT-rtPCR-positive at only one visit. On multivariable analysis, individuals with symptoms, people living with HIV with higher HIV viral load (≥400 copies per mL; *vs* uninfected HIV status), and individuals with a low minimum Ct value (≤30) shed SARS-CoV-2 RNA for longer (table 3). Positive serology before the episode was associated with decreased duration of shedding.

**Table 3:** Factors associated with duration of SARS-CoV-2 viral RNA positivity in 662 episodes of infection

	**Viral RNA shedding duration, days, mean (SD; range)**	Univariate HR*(95% CI)	Multivariable adjusted HR*(95% CI)
**Age group, years**			
<5	11·6 (6·8; 4–35)	1 (ref)	..
5–12	10·9 (7·4; 4–52)	1·1 (0·8–1·4)	..
13–18	11·5 (7·8; 4–60)	1·0 (0·7–1·3)	..
19–39	11·8 (12·7; 4–137)	0·8 (0·6–0·9)	..
40–59	11·8 (7·1; 4–43)	1·0 (0·7–1·3)	..
≥60	13·2 (9·0; 4–52)	0·7 (0·5–0·9)	..
**Sex**			
Female	12·2 (10·1; 4–137)	0·8 (0·7–0·9)	..
Male	10·6 (6·7; 4–52)	1 (ref)	..
**HIV status and viral load copies per mL**†			
Uninfected	11·3 (7·5; 4–60)	1 (ref)	1 (ref)
Infected <400 copies	11·7 (7·3; 4–52)	1·0 (0·7–1·2)	0·9 (0·7–1·1)
Infected ≥400 copies	18·5 (26·6; 4–137)	0·3 (0·2–0·5)	0·4 (0·3–0·6)
HIV status or viral load unknown	8·9 (5·6; 4–33)	1·5 (1·1–2·2)	1·3 (0·9–1·9)
**HIV status and CD4 T-cell count per μL**†			
Uninfected	11·3 (7·5; 4–60)	1 (ref)	..
Infected ≥200 CD4 cells	11·9 (8·4; 4–52)	0·9 (0·7–1·1)	..
Infected <200 CD4 cells	30·2 (43·5; 4–137)	0·1 (0·1–0·3)	..
HIV status or CD4 cell count unknown	9·2 (5·6; 4–33)	1·5 (1·1–2·1)	..
**Other underlying illness**‡			
Absent	11·6 (9·1; 4–137)	1 (ref)	..
Present	12·0 (7·0; 4–34)	1·0 (0·8–1·4)	..
**BMI**§			
Underweight	12·9 (10·8; 4–60)	0·8 (0·6–1·1)	..
Normal weight	11·2 (9·6; 4–137)	1 (ref)	..
Overweight	12·3 (7·6; 4–46)	1·0 (0·8–1·2)	..
Obese	11·6 (7·5; 4–43)	1·0 (0·8–1·2)	..
**COVID-19-related symptoms**			
Absent	11·0 (7·5; 4–60)	1 (ref)	1 (ref)
Present	14·8 (14·7; 4–137)	0·6 (0·5–0·7)	0·7 (0·6–0·9)
**Minimum cycle threshold value**			
≤30	12·8 (9·3; 4–137)	0·3 (0·3–0·4)	0·3 (0·3–0·4)
>30	6·0 (4·3; 4–28)	1 (ref)	1 (ref)
**Seropositive before episode**			
No	12·3 (9·3; 4–137)	1 (ref)	1 (ref)
Yes	7·9 (5·5; 4–36)	2·0 (1·6–2·5)	1·3 (1·1–1·7)
**SARS-CoV-2 vaccine status**			
Unvaccinated	11·5 (9·1; 4–137)	1 (ref)	..
Partially vaccinated	15·3 (7·9; 4–29)	0·7 (0·4–1·4)	..
Fully vaccinated	12·1 (6·7; 4–31)	1·0 (0·7–1·3)	..
**Variant**			
Wuhan-Hu-1	10·8 (7·5; 4–43)	1 (ref)	1 (ref)
Beta	13·0 (11·5; 4–137)	0·7 (0·5–0·9)	1·0 (0·5–2·2)
Alpha	12·0 (7·7; 4–22)	0·9 (0·4–1·9)	0·8 (0·6–1·1)
Delta	10·6 (6·5; 4–52)	1·1 (0·8–1·4)	1·1 (0·8–1·5)

The analysis was restricted to 662 episodes of real-time RT-PCR-confirmed SAR-CoV-2 infection with onset more than 14 days after the start of follow-up. Samples were collected at 3–4 day intervals, thus values of 4 days represent a single positive swab. HR=hazard ratio. BMI=body-mass index.

* Estimated with Weibull accelerated failure time regression adjusted for clustering by site and household; HR<1 corresponds to prolonged duration of viral RNA shedding.

† HIV data available for 629 (95·0%) of 662 individuals eligible for this analysis; among 94 people living with HIV eligible for this analysis, 93 (98·9%) had available data on HIV viral load and 91 (96·8%) had available data on CD4 T-cell count.

‡ Self-reported history of asthma, lung disease, heart disease, stroke, spinal cord injury, epilepsy, organ transplant, immunosuppressive therapy, organ transplantation, cancer, liver disease, renal disease, or diabetes.

§ Calculated with the formula (weight in kg)/(height in m^2^); underweight (age <18 years) was defined as weight for age or BMI <–2 SDs of the WHO Child Growth Standards; underweight (age ≥18 years) as BMI <18·5kg/m^2^; overweight (age <18 years) as BMI >+1 and ≤+2 SDs of the WHO Child Growth Standards; overweight (age ≥18 years) as BMI ≥25 kg/m^2^ and <30 kg/m^2^; obese (age <18 years) as BMI >+2 SDs of the WHO Child Growth Standards; and obese (age ≥18 years) as BMI ≥30 kg/m^2^.

Among 195 households with at least one RT-rtPCR-confirmed SARS-CoV-2 infection cluster, 72 (36·9%) had one cluster during follow-up, 83 (42·6%) had two clusters, 31 (15·9%) had three clusters, and nine (4·6%) had more than three clusters (total of 369 clusters). 336 clusters were recorded more than 14 days after the start of follow-up; mean cluster duration among these was 15·6 days (SD 12·9; range 4–137). We included 180 clusters from 101 households for analysis of HCIR. In this subset of households, HCIR was 23·9% (195 of 817 susceptible household members infected [95% CI 19·8–28·4]). HICIR was 23·3% (20 of 86 [17·3–30·5]) for symptomatic index cases and 23·9% (175 of 731 [20·1–28·3]) for asymptomatic index cases (table 4). On multivariable analysis, low minimum Ct value (≤30) of the index case, index case female sex, index case age younger than 5 years and age 40–59 years (*vs* ≥60 years), household contact age 5–18 years (*vs* 19–39 years), and infection with beta or delta variant (*vs* Wuhan-Hu-1) were associated with increased HCIR (table 4). The mean generation interval was 7·5 days (SD 4·8) (range 2–21 days after excluding outliers; appendix p 34). On multivariable analysis, generation interval was shorter if the index case was symptomatic, and longer if the index case shed virus for longer (>4 days *vs* ≤4 days) or was infected with alpha, beta or delta variant (*vs* wild-type; appendix pp 21–22).

**Table 4:** Factors associated with HCIR among 180 clusters in 101 households

		HCIR*,**n/N (%)**	**Univariate OR**†**(95% CI)**	**Multivariable adjusted OR**†**(95% CI)**
**Characteristics of the index case**				
Age group, years				
	<5	18/54 (33·3%; 20·8–48·7)	7·1 (1·9–27·1)	6·7 (1·6–28·1)
	5–12	42/163 (25·8%; 19·3–33·4)	2·6 (0·8–8·3)	2·0 (0·6–6·8)
	13–18	38/169 (22·5%; 17·7–28·1)	1·9 (0·6–6·0)	1·7 (0·5–5·6)
	19–39	48/247 (19·4%; 14·7–25·2)	2·2 (0·7–6·6)	2·5 (0·8–8·2)
	40–59	41/115 (35·7%; 24·4–48·8)	5·9 (1·7–19·9)	5·3 (1·5–19·4)
	≥60	8/69 (11·6%; 5·9–21·6)	1 (ref)	1 (ref)
Sex				
	Female	124/469 (26·4%; 22·0–31·4)	1·7 (1·0–2·8)	1·9 (1·1–3·4)
	Male	71/348 (20·4%; 15·3–26·6)	1 (ref)	1 (ref)
HIV status and viral load copies per mL				
	Uninfected	152/646 (23·5%; 20·1–27·3)	1 (ref)	..
	Infected <400 copies	31/105 (29·5%; 21·9–38·5)	1·1 (0·6–2·3)	..
	Infected ≥400 copies	5/27 (18·5%; 6·9–41·2)	0·7 (0·2–2·8)	..
	HIV status or viral load unknown	7/39 (17·9%; 8·2–34·8)	1·0 (0·3–3·6)	..
HIV status and CD4 T-cell count per μL				
	Uninfected	152/646 (23·5%; 20·1–27·3)	1 (ref)	..
	Infected ≥200 CD4 cells	30/113 (26·5%; 20·1–34·1)	1·0 (0·5–2·0)	..
	Infected <200 CD4 cells	5/15 (33·3%; 9·6–70·1)	1·3 (0·3–6·7)	..
	HIV status or CD4 cell count unknown	8/43 (18·6%; 8·8–35·0)	1·1 (0·3–3·3)	..
BMI§				
	Underweight	18/63 (28·6%; 17·7–42·7)	1·1 (0·5–2·7)	..
	Normal weight	85/397 (21·4%; 17·4–26·1)	1 (ref)	..
	Overweight	38/173 (22·0%; 17·4–27·3)	1·1 (0·6–2·2)	..
	Obese	54/184 (29·3%; 20·2–40·5)	2·4 (1·2–4·6)	..
Symptoms				
	Absent	175/731 (23·9%; 20·1–28·3)	1 (ref)	..
	Present	20/86 (23·3%; 17·3–30·5)	1·0 (0·5–2·0)	..
Duration of viral RNA shedding, days				
	≤4	15/170 (8·8%; 5·9–12·9)	1 (ref)	..
	>4	180/647 (27·8%; 23·5–32·6)	4·4 (2·2–9·1)	..
Minimum Ct value				
	≤30	182/659 (27·6%; 23·5–32·1)	5·8 (2·6–12·8)	5·3 (2·3–12·4)
	>30	13/158 (8·2%; 4·8–13·8)	1 (ref)	1 (ref)
Epidemic wave				
	1	14/155 (9·0%; 5·5–14·5)	1 (ref)	..
	2	74/328 (22·6%; 17·0–29·4)	3·2 (1·4–7·4)	..
	3	107/334 (32·0%; 27·7–36·8)	9·8 (4·2–23·2)	..
Variant				
	Wuhan-Hu-1	13/146 (8·9%; 5·2–14·8)	1 (ref)	1 (ref)
	Alpha	4/7 (57·1%; 17·8–89·1)	17·7 (0·8–400·2)	20·0 (0·9–433·6)
	Beta	73/335 (21·8%; 16·2–28·7)	3·2 (1·4–7·4)	3·3 (1·4–8·2)
	Delta	105/329 (31·9%; 27·7–36·4)	9·8 (4·0–23·8)	10·4 (4·1–26·7)
Site				
	Rural	101/494 (20·4%; 16·7–24·8)	1 (ref)	..
	Urban	94/323 (29·1%; 22·7–36·3)	1·5 (0·8–2·7)	..
**Characteristics of the household contact**				
Age group, years				
	<5	23/105 (21·9%; 14·4–31·9)	1·3 (0·6–2·8)	1·2 (0·6–2·7)
	5–12	69/265 (26·0%; 18·4–35·4)	2·0 (1·1–3·6)	2·0 (1·1–3·8)
	13–18	35/112 (31·3%; 24·2–39·3)	2·8 (1·4–5·7)	3·1 (1·4–6·7)
	19–39	29/165 (17·6%; 12·0–25·0)	1 (ref)	1 (ref)
	40–59	23/92 (25·0%; 17·9–33·8)	1·6 (0·8–3·5)	2·0 (0·9–4·7)
	≥60	16/78 (20·5%; 13·7–29·6)	1·2 (0·5–2·8)	1·4 (0·6–3·6)
Sex				
	Female	120/481 (24·9%; 21·4–28·8)	1·3 (0·9–2·0)	..
	Male	75/336 (22·3%; 16·9–28·9)	1 (ref)	..
HIV status and viral load copies per mL				
	Uninfected	164/679 (24·2%; 19·1–30·0)	1 (ref)	..
	Infected <400	15/77 (19·5%; 11·5–31·0)	0·8 (0·4–1·7)	..
	Infected ≥400	8/21 (38·1%; 20·7–59·3)	1·9 (0·6–6·3)	..
	HIV status or viral load unknown	8/40 (20·0%; 9·2–38·1)	0·6 (0·2–1·7)	..
HIV status and CD4 T-cell count per μL				
	Uninfected	164/679 (24·2%; 19·1–30·0)	1 (ref)	..
	Infected ≥200 CD4 cells	22/90 (24·4%; 15·9–35·7)	1·0 (0·5–2·0)	..
	Infected <200 CD4 cells	1/8 (12·5%; 6·2–76·3)	0·6 (0·1–7·1)	..
	HIV status or CD4 cell count unknown	8/40 (20·0%; 9·2–38·1)	0·6 (0·2–1·7)	..
Other underlying illness				
	Absent	179/730 (24·5%; 19·9–29·8)	1 (ref)	..
	Present	16/87 (18·4%; 12·1–26·9)	0·6 (0·3–1·2)	..

Data are n/N (%; 95% CI), unless otherwise stated. Additional factors evaluated but not found to be statistically significant include SARS-CoV-2 vaccination status of the index case, vaccination status of the household contact, underlying tuberculosis of the index case or household contact, BMI of the contact, alcohol use or smoking status of the index case or household contact, fuel used for cooking, water source for handwashing, number of people in the household, household crowding, and individual symptoms in the index case including cough, fever, difficulty breathing, runny nose, and other symptoms. HCIR=household cumulative infection risk. OR=odds ratio. BMI=body mass index. Ct=cycle threshold. RT-rtPCR=real-time RT-PCR.

* HCIR was defined as the probability of secondary infections within a household after SARS-CoV-2 introduction and estimated by dividing the number of subsequent individuals with confirmed infection within a household cluster (n) after SARS-CoV-2 introduction by the number of susceptible (no previous infection on RT-rtPCR or serology) household members (N); denominator (N) is the sum of all exposed susceptible individuals for all clusters including multiple clusters and accounting for the fact that in subsequent clusters some household contacts might no longer be considered susceptible.

† Estimated with logistic regression adjusted for clustering by site and household.

§ Calculated with the formula (weight in kg)/(height in m^2^); underweight (age <18 years) was defined as weight for age or BMI <–2 SDs of the WHO Child Growth Standards; underweight (age ≥18 years) as BMI <18·5kg/m^2^; overweight (age <18 years) as BMI >+1 and ≤+2 SDs of the WHO Child Growth Standards; overweight (age ≥18 years) as BMI ≥25 kg/m^2^ and <30 kg/m^2^; obese (age <18 years) as BMI >+2 SDs of the WHO Child Growth Standards; and obese (age ≥18 years) as BMI ≥30 kg/m^2^.

## Discussion

Using intensive systematic repeated sampling among household cohorts in two largely unvaccinated South African communities, we found that by Aug 28, 2021, 62·4% of individuals had been infected with SARS-CoV-2. 11·6% of individuals experienced at least one repeat episode of infection within 13 months of follow-up. Only 14·7% of infections were associated with symptoms; of these, 9·3% (n=9) individuals were hospitalised and 2·1% (n=2) died. SARS-CoV-2 delta variant infections were more likely to be symptomatic compared with Wuhan-Hu-1 strain infection. In households with at least one SARS-CoV-2 infection, SARS-CoV-2 was transmitted to 23·9% of household contacts irrespective of symptoms in the index case. Index and household contact age, index case female sex, and infecting SARS-CoV-2 variant, and index case RNA viral load were the main predictors of onward transmission. The odds of household transmission showed a 3-times increase with the beta variant, and a 10-times increase with the delta variant.

Previous studies have generated wide-ranging estimates of the proportion of SARS-CoV-2 infections that are asymptomatic. A recent systematic review found that 20% (95% CI 17–25; prediction interval 3–67) of SARS-CoV-2 infections remained asymptomatic throughout infection and that transmission was lower from asymptomatic individuals than symptomatic individuals.^18^ We found that 85·3% of infections were asymptomatic, 92·5% among individuals younger than 19 years and 76·1% among individuals aged 19 years or older, despite active symptom evaluation twice a week. Three US-based cohort studies implementing PCR testing once a week for SARS-CoV-2, irrespective of symptoms, plus additional testing with symptomatic illness, found asymptomatic fractions of 31% in a community-based household cohort including children, 35% in pregnant women, and 11% among health-care workers,^19^, ^20^, ^21^ which are lower than the estimates in our study. The lower asymptomatic proportion in these studies could be because swabbing twice a week allows identification of transient asymptomatic illness, or because the symptomatic fraction could be truly lower in the populations included in our study. Compared with other studies, our study population was young, with only 8·6% of individuals aged 60 years or older, reflecting the general South African population.^3^ Despite the low overall symptomatic fraction, symptomatic fraction increased with age, in accordance with previous studies.^18^, ^22^

A systematic review and meta-analysis of 87 household transmission studies of SARS-CoV-2 reported an estimated secondary attack rate of 18·9% (95% CI 16·2–22·0),^23^ slightly lower than our estimate of 23·9%. The review also reported that household secondary attack rates were higher from symptomatic index cases, that adults were more likely than children to acquire infection, and that male or female sex was not associated with transmission. Previous studies have found that viral load and duration of SARS-CoV-2 shedding are reduced among mild and asymptomatically infected individuals compared with individuals with severe illness.^24^ Although we did not find that symptom profile was associated with risk of transmission, we did find that elevated index case viral RNA load was associated with increased frequency of transmission, similar to previous studies.^23^ We also found female participants were more likely to transmit SARS-CoV-2, in contrast to the previous systematic review, potentially reflecting closer contact with other household members in our study population. Several studies have reported increased transmissibility of the beta and delta SARS-CoV-2 variants.^6^, ^25^ We found the odds of household transmission were approximately 3-times higher with beta variant and 10-times higher with delta variant compared with Wuhan-Hu-1 infections. It is possible that participants changed their behaviour once informed they were infected with SARS-CoV-2, leading to reduced HCIR; however, data on behaviour after a SARS-CoV-2 diagnosis were not available.

Although attack rates were low in children younger than 5 years, this age group was more likely to transmit SARS-CoV-2 than the oldest age group (≥60 years). Symptomatic fraction was lowest in children and adolescents younger than 18 years. Indviduals aged 13–18 years had the highest attack rates and were most likely to acquire infection within the household and were most likely to be reinfected. Previous studies have found low attack rates and symptomatic fraction in children (although relatively increased in age group 13–18 years, similar to our finding), and that children are less likely to transmit SARS-CoV-2 and have reduced susceptibility to infection.^23^, ^26^ The delta variant has been associated with increased attack rates in children and adolescents compared with other variants in South Africa and elsewhere.^27^ In several countries, the relative increase in incidence in children and adolescents has in part been attributed to a shift in the age distribution of cases as vaccination expanded to adult age groups.^28^ In South Africa, where vaccination coverage is low, infection rates were higher among adults in the first two waves, potentially contributing to higher attack rates in children and adolescents with delta variant. Differences in circulating variants over time and geographic location might have contributed to differences in the contribution of adolescents to transmission in previous studies.^27^ In addition, most previous studies did not include systematic longitudinal RT-rtPCR testing irrespective of symptoms in children, adolescents, and adults, potentially biasing against detection of minimally symptomatic infections in children.

Previous studies have found that people living with HIV have increased likelihood of being hospitalised, and that hospitalised people living with HIV without HIV viral suppression shed SARS-CoV-2 at a high viral load for longer than HIV-uninfected individuals, but data from community settings are scarce.^7^, ^29^, ^30^ We found that people living with HIV who were not virally suppressed were more likely to develop symptomatic illness when infected with SARS-CoV-2, and shed SARS-CoV-2 for longer, when compared with HIV-uninfected individuals, potentially contributing to the evolution of novel variants of SARS-CoV-2. Other studies have found obesity associated with COVID-19 infection, possibly because of immune dysfunction or increased ACE2 expression.^31^

Our study had several strengths. Participating households were randomly sampled from a rural and an urban community and followed up for 13 months during three waves of SARS-CoV-2 infection in South Africa. Participating individuals were sampled twice a week, irrespective of symptoms, allowing for improved accuracy in ascertainment of SARS-CoV-2 burden, symptomatic fraction, and transmission from asymptomatic individuals. Our unique study design combining frequent RT-rtPCR and serological testing also allowed for improved ascertainment of infection burden.

Our study also had several limitations. We included two communities and households with more than two members, potentially limiting generalisability of study findings. The finding of a higher attack rate and reinfection rate in the urban community could be a result of greater population density. We might have introduced selection bias because individuals who did not agree to participate in the study and those excluded because of fewer than ten follow-up visits might have differed from included participants. Participants were sampled with midturbinate nasal swabs because of potential SARS-CoV-2 transmission risk with collection of more sensitive nasopharyngeal swab specimens. This sampling method could have led to some missed infections. However, the strong association between RT-rtPCR-confirmed and serology-confirmed infection in individuals with both specimen types available suggests that most infections were detected. Repeated questioning on symptoms twice a week might be associated with participant fatigue and under-reporting. We implemented several measures to reduce this potential bias including weekly retraining of field workers on symptom collection and regular field supervisory visits to evaluate data collection and symptom recording. A study of influenza infection in the same population with a similar study design found that 268 (56·1%) of 478 individuals infected with influenza were symptomatic, suggesting the robustness of our data.^9^ We did not quantify viral RNA load but instead this measure was inferred, using Ct value as a proxy. We assumed that all secondary cases acquired infection within the household, but infection could have been acquired outside the household, potentially leading to overestimation of HCIR.

In conclusion, we found a high rate of SARS-CoV-2 infection in households in a rural and an urban community in South Africa, with most infections being asymptomatic in individuals of all ages. Individuals aged 13–18 years had the highest attack rates and were more likely to acquire infection than other age groups. The HCIR was 23·9% and did not differ by presence of symptoms in index cases, but, accounting for age and sex, was increased in households where the index case had a high RNA viral load, or in households with beta or delta variant infection. Asymptomatic individuals transmitted SARS-CoV-2 at a similar rate to symptomatic individuals suggesting that interventions targeting symptomatic individuals, such as promotion of community testing and contact tracing of individuals tested because they report symptoms, might have limited effects in this setting. Shortly after conclusion of the study, the emergence of the omicron variant caused rapid growth in case incidence in the study areas and nationally, likely to be due to a combination of immune escape and increased transmission characteristics.^32^ Future studies should address the build up of cross-protective immunity after successive waves of infection in settings with high attack rates, and the consequences for future epidemic resurgences.

## Data sharing

The investigators welcome enquiries about possible collaborations and requests for access to the data set. Data (including individual participant data and a data dictionary defining each field in the set) will be shared after approval of a proposal and with a signed data access agreement. Investigators interested in more details about this study, or in accessing these resources, should contact the principle investigator and corresponding author, Cheryl Cohen (cherylc@nicd.ac.za). The full study protocol including informed consent forms can be found on the South Africa National Institute for Communicable Diseases (NICD) website.

## Declaration of interests

CC has received grant support from Sanofi Pasteur, the US Centers for Disease Control and Prevention (CDC), Wellcome Trust, the Programme for Applied Technologies in Health (PATH), Bill & Melinda Gates Foundation, and the South African Medical Research Council (SA-MRC). AvG has received grant support from the US CDC, the African Society for Laboratory Medicine and Africa Centres for Disease Control and Prevention, the SA-MRC, WHO Regional Office for Africa, the Fleming Fund, WHO, and Wellcome Trust. NW reports grants from Sanofi Pasteur and the Bill & Melinda Gates Foundation. NAM has received a grant to his institution from Pfizer to conduct research in patients with pneumonia. JM has received grant support from Sanofi Pasteur and PATH. JB has received grant funds from the European and Developing Countries Clinical Trials Partnership, the Fleming Fund, and the SA-MRC. All other authors declare no competing interests.
