# Supplementary material for: SARS-CoV-2 incidence, transmission, and reinfection in a rural and an urban setting: results of the PHIRST-C cohort study, South Africa, 2020–21
Source: Lancet Infect Dis. 2022 Jun;22(6):821–34. doi: 10.1016/S1473-3099(22)00069-X (PMC8920516; doi:10.1016/S1473-3099(22)00069-X)
Supplement: Supplementary appendix [file mmc1.pdf]

THE LANCET
Infectious Diseases

Supplementary appendix

This appendix formed part of the original submission and has been peer reviewed. We post it as supplied by the authors.

Supplement to: Cohen C, Kleynhans J, von Gottberg A, et al. SARS-CoV-2 incidence, transmission, and reinfection in a rural and an urban setting: results of the PHIRST-C cohort study, South Africa, 2020–21. *Lancet Infect Dis* 2022; published online March 14. https://doi.org/10.1016/S1473-3099(22)00069-X.

Supplement to - SARS-CoV-2 incidence, transmission and reinfection in South Africa

Supplemental methods

Household selection and participant enrolment

We randomly selected households using different methods in each site. In Agincourt, for the original "A Prospective Household cohort study of Influenza, Respiratory Syncytial virus and other respiratory pathogens community burden and Transmission dynamics in South Africa (PHIRST) study", in 2017 and 2018 we selected two villages out of 29 within the Health and Demographic Surveillance Site (HDSS) according to convenience. Within these villages, households with >2 members were randomly selected from an annually updated list of all enumerated households within the HDSS using simple random sampling. Using a sampling frame of all enumerated households, we restricted to households with >2 household members. We then used a computer-generated algorithm to randomly select households. We only included households with >2 household members as smaller households could provide limited information on household transmission. For the current study, we approached all houses participating in the initial PHIRST study in 2017 and 2018 and if sufficient numbers were not enrolled we approached additional households from the same villages using the same sampling frame and ensuring similar total numbers of included households from all four villages. We did not approach houses participating in PHIRST in 2016 at the rural site because logistically it was more feasible to conduct the study in four (rather than six) villages.

In Jouberton Township, for the original PHIRST study, a list of 450 random global positioning system (GPS) coordinates were generated in the study area using Google Earth as previously described.[3] Study staff navigated to the coordinates and selected the nearest house within 30 meters of the location. If there was no dwelling within 30m the coordinates were discarded. We approached all houses participating in the initial PHIRST study from 2016 through 2018 and if sufficient numbers were not enrolled we approached additional households from the same township using the same sampling frame until the desired sample size was reached. We approached houses participating in PHIRST from all three years (2016-2018) at the urban site because logistically this was feasible due to the compact size and random distribution of enrolled households.

Detailed descriptions of the two sites and included households have been published previously.[1,2] Households in the rural site were more likely to have children aged <5 years living in the house, more likely to use wood as cooking fuel (vs electricity), have a toilet apart from the main dwelling and less likely to have secure water supply or place for handwashing. The rural site had mainly unpaved road which contributed to dust. Use of wood for cooking and burning of domestic waste

because of absent waste removal services also contributed to air pollution at the rural site. Number of rooms in houses and numbers of household members were similar between the sites.

Household size was similar for included houses and general community at the rural site (number of household members=4, interquartile range 1-20 in study vs n=4 interquartile range 1-14 in community) and at the urban site (number of household members=5, interquartile range 3-14 in study vs n=4 interquartile range 3-5 in community)[4].

Sample size

We aimed to study infection and reinfection in a total of 1000 individuals. Assuming an average household size of 5 individuals and loss to follow up of 10%, we planned to enrol approximately 110 households from each site. Assuming a constant community attack rate of 60% across 5 selected age groups (based on an estimated R_0 value of 2.5[5]) for a 95% confidence interval, a 10% desired absolute precision and a 1.3 design (household cluster) effect, 120 individuals were needed in each age stratum. The expected age distribution in the target cohort was 16% for individuals aged <5 years (160 expected individuals in the cohort), 41% for individuals aged 5-18 years (410 expected individuals in the cohort), 26% for individuals aged 19-44 years (260 expected individuals in the cohort), 12% for individuals aged 45-64 (120 expected individuals in the cohort) and 5% for individuals aged ≥65 years (50 expected individuals in the cohort).

We assumed 30% symptomatic fraction among individuals infected with SARS-CoV-2.[6] We assumed 20% severe cases and a 2% mortality rate among all symptomatic individuals. The sample sizes to estimate the above parameters for a 95% confidence interval and a 10% desired absolute precision are as follows: (i) 30% symptomatic fraction among infected individuals: 81 infected individuals; (ii) 20% severe cases among symptomatic individuals: 62 symptomatic individuals severe; (iii) 2% mortality among symptomatic individuals: 8 symptomatic individuals died. A total of 270 individuals with SARS-CoV-2 infection (i.e., 81 symptomatic individuals/0.3 symptomatic fraction; community attack rate: 27.0%) will be needed to estimate the above-mentioned parameters with the desired level of precision.

Staff and participant safety

A number of measures were introduced to minimise risks to the study team and potentially to participants due to the circulation of SARS-CoV-2. Front-line staff including study nurses were trained in infection control procedures including proper hand hygiene and the correct use of surgical face masks, not only to minimize their own risk of infection when in close contact with patients during home visits and elsewhere, but also to minimize the risk of the nurses acting as a vector of infection between household members or between households. Staff were required to wear gloves for collection of patient specimens. All staff were offered influenza vaccination prior to commencing the study, and again before the influenza season in 2021. Staff were properly trained in collection of blood specimens and disposal of sharps. Staff were requested to stay home from work if they had symptoms of respiratory illness or if they had been in contact with a confirmed of suspected SARS-CoV-2 case without appropriate personal protective equipment. Retraining of all safety procedures including field implementation evaluation was performed weekly. Additional measures implemented included use of dedicated staff transport adhering to government regulations on occupancy numbers, provision of scrubs, headcover, aprons and surgical masks for fieldwork, consistent physical distancing for all staff, frequent disinfection of all equipment between households, use of hand sanitiser and daily temperature checks. All interviews were conducted from a distance of at least 2 metres from the interviewer outside of the house if possible and with no physical contact between participants and study staff member. The study convened a safety advisory board (SAB) to advise investigators on the safety procedures in the study including the appropriate PPE, safety procedures for interaction with infected household members, other workplace safety concerns. The SAB members were independent of the study and constituted clinical, microbiology and infection prevention and control experts. A community representative was included from each site.

Individuals testing positive for SARS-CoV-2 were notified through the NMC (Notifiable Medical Conditions) system and rapidly communicated to the provincial Departments of Health (Mpumalanga and North West provinces) and contact tracing was performed by local public health authorities. Participants were advised to isolate if they tested positive for SARS-CoV-2 or if they screened positive for COVID-like symptoms whilst awaiting results. As per National guidelines, isolation was advised for 10 days from the onset of symptoms or from the date of testing in the case of an asymptomatic case.

Nurses counselled participants to isolate in a separate room of the house, to limit contact with other household members, to wear a mask when leaving their isolation room to use the bathroom, to wipe down items used in the common space (such as taps or toilet), to not share food, and to sanitise regularly. The household was also advised to ensure good ventilation within the house (opening windows and doors).

Due to the nature of the rural and urban environment, the adoption of these measures was adversely affected by numerous factors. Those without enough rooms attempted to lower transmission risk by minimising the number of people sleeping in the same room, limiting the time spent in the same room as the index case, having the index case wear a mask whilst with household members, and opening windows. Where a child was the index case, the mother or primary caregiver would stay with the child to care for them. Many found it difficult to limit the movement of, and interactions with, SARS-CoV-2 positive children especially their interactions with other children in the household. Many participants were asymptomatic during their SARS-CoV-2 infection and this combined may have negatively impacted adherence to recommended measures. The average turn-around time for samples from collection to returning result to the participant was 5 days for symptomatic individuals and 10-14 days for asymptomatic individuals leading to delays in implementation of recommended measures.

A nasopharyngeal swab was collected from all field workers on a weekly basis and tested for SARS-CoV-2 to document possible infection transmission to and by fieldworkers. Any positive test was considered an adverse event and were reported to the study SAB.

Participant enrolment

In the enrolment visit, field staff first confirmed that the household contained >2 members and then requested permission from the head of household to inform members about the study. After a minimum of three failed attempts, if the head of household was unavailable or a minor, the household was excluded. Study staff requested informed consent to participate in the study from all household members aged ≥18 years, assent from children aged 7 to 17 years, and consent from a parent or guardian for children younger than 18 years. If a household withdrew their participation while in the first 6 months of the study follow up, they were replaced with a newly recruited household.

Data collection

Symptom data collection and temperature measurement were performed at each follow up visit. Symptoms collected included fever (self-reported or measured tympanic temperature ≥38°C), cough, difficulty breathing, sore throat, nasal congestion, vomiting, diarrhoea, abdominal pain or loss of smell or taste, muscle aches, fatigue, headache and confusion. Symptom data collected differed by age group to account for age-specific differences in clinical presentation and difficulty collecting information on subjective symptoms such as muscle aches or headache from young children. Symptoms collected in each age group are detailed in supplementary table 1. Symptom data for children was consistently collected from the individual identified as primary caregiver at the time of

enrolment. For older children and adolescents, self-reported symptom data were also accepted. Field workers received refresher training on different aspects of study implementation including data collection (with specific focus on respiratory sign and symptom data), specimen collection and use of online databases at least weekly. Field workers were encouraged to observe participants for visible symptoms such as cough and prompt for symptoms if not reported. Measured tympanic temperature was recorded at each study visit and either reported or measured fever was included as presence of fever. Site supervisors conducted regular (at least monthly) supervisory visits to assess study implementation in the field and external (teams from outside the study sites) supervisory visits were conducted quarterly. During the two weeks in December over the Christmas and New Year holidays, and again at Easter home visits for collection of mid-turbinate nasal swabs were conducted only once a week instead of twice-weekly.

Written vaccination history was obtained for all children aged <5 years from patient-held immunisation records and, if needed, vaccination records at health facilities. Primary caregivers giving a history of the child never being vaccinated were recorded as unvaccinated.

Vaccination against SARS-CoV-2 commenced in South Africa in February 2021, initially among health care workers using the Johnson & Johnson (J&J) vaccine as part of a Phase 3 study, followed by the initiation of national vaccination programme including the Pfizer BioNTech and J&J vaccines in individuals ≥60 years of age from May 2021, sequentially moving to 50-59 year olds and 35-49 year olds by the end of follow up. Only the J&J and Pfizer vaccines were available during the study period.

Household income was evaluated through self-reporting by the head of household rounded to the nearest rand. For households where income varied from month to month, the head of household was asked to provide the average monthly household income over the previous 12 months.

HIV status was obtained from patient medical records if a participant reported being HIV-infected, or by nurse-administered rapid HIV test with pre- and post-test counselling for participants with unknown, or self-reported HIV-negative status. Patients newly diagnosed with HIV were referred to the nearest facility for assessment and initiation of antiretroviral treatment. Infants were defined as HIV exposed but uninfected if they were HIV-uninfected but the mother was living with HIV. For people living with HIV (PLHIV), samples were collected for determination of CD4 T-lymphocyte count and HIV quantitative viral load testing at diagnosis or enrolment and current use of antiretroviral therapy was documented.

Laboratory methods

All specimens testing SARS-CoV-2-rRT-PCR-positive were confirmed by repeat testing of a second aliquot, and PCR testing in duplicate. Specimens testing positive on at least one duplicate were considered positive. If a specimen was confirmed positive after repeat testing, the results [C_t value and targets testing positive] from the first positive test were included in the analysis. For the first 46 visits this testing was done retrospectively after 1 to 6 months of storage at -70 degrees centigrade. Following this, confirmatory resting was performed in real time following identification of a rRT-PCR-positive sample.

We confirmed presence of human DNA in samples through testing for the RNaseP gene on a random 10% of samples weekly until 17 March 2021 and subsequently tested all samples for presence of RNaseP. Of 13581 samples tested for RNaseP, human DNA was detected in 13122 (97%).

All confirmed positive samples were tested to identify variants of concern using the Allplex™ SARS-CoV-2 Variants I assay (Seegene Inc., Seoul, Korea). This assay targets the RdRp gene, HV69/70 deletion, N501Y and E484K mutations, thus identifying the B.1.351/P1 (Beta/Gamma) and B.1.1.7 (Alpha) variants. From May 2021 SARS-CoV-2-positive samples were also tested using the Allplex™ Variants II assay (Seegene Inc., Seoul, Korea) which detects the L452R mutation (Delta) and differentiates Beta (K417N) from Gamma (K417T).

For sequencing of SARS-CoV-2, we used AmpliSeq for SARS-CoV-2 (Illumina), which is based on the ARTIC SARS-CoV-2 sequencing protocol (https://www.protocols.io/view/illumina-nextera-dna-flex-library-construction-and-bhjgj4jw), on the Ion Torrent Genexus platform. Genomes were assembled using the Exatype SARS-CoV-2 pipeline (https://sars-cov-2.exatype.com/), which includes de-duplicating sequenced reads data, base-calling, de-multiplexing, removal of amplicon primer sequences prior to variant calling, mapping to a reference, polishing and finally consensus sequence generation. Clade and lineage assignments were made using the online Nextclade (https://clades.nextstrain.org/) and Pangolin (https://pangolin.cog-uk.io/) applications, which also enable identification of known variants of concern as well as novel mutations. We used in-house R codes and AliView (https://ormbunkar.se/aliview/) to polish sequences and display analyses. In addition we used a Galaxy SARS-CoV-2 pipeline to analyse minority variant analysis.

Definitions and statistical analyses

We included individuals with ≥10 completed follow-up visits. If an individual was excluded from analysis because of insufficient follow-up visits and this resulted in <70% of household members being included in the study than the household was dropped from the analysis, else the household was retained. In practice, no household was dropped as a result of an individual member having insufficient follow up visits.

We defined wave timing based on epidemic timing in the two communities. The cut off between waves was the first follow up visit with the lowest number of new positive participants between peaks. This was done separately by site. First wave episodes or clusters were defined as having onset before 19 December 2020 at both sites, second wave as having onset before 9 June 2021 in Agincourt and 23 June 2021 in Klerksdorp, and third wave as onset up to 28 August at both sites when intense follow-up ended.

For assessment of variants, data from both the Allplex™ SARS-CoV-2 Variants I and II assay and sequencing were used.

Variants were determined based on:

1. At sample level, each sample was characterised using AmpliSeq for SARS-CoV-2 (Illumina) full genome sequencing and Allplex™ SARS-CoV-2 Variants I and II PCR assays.
2. Variants were assigned firstly based on sequencing results where sequence coverage was good enough to allow clade and lineage assignments (using the online Nextclade (https://clades.nextstrain.org/) and PANGOLin (https://pangolin.cog-uk.io/) applications, which also enable identification of known variants of concern as well as novel mutations.).
3. Where sequence coverage was too low for classification, or where sequencing was not performed due to initial diagnostic rRT-PCR Ct values >35, variant rRT-PCR results were used. Where no variant-specific mutations were detected in the rRT-PCR, and the RdRp gene Ct was <35, and internal controls for both assays were detected, the episodes were classified as attributed to wild-type virus Where no variant-specific mutations were detected in the rRT-PCR, but the RdRp gene Ct was ≥35, variant for the sample was left as unassigned.

4. Any discrepancies identified between sequencing and rRT-PCR results were verified by re-extracting the original sample and repeating both sequencing and variant rRT-PCR assay results. If results were still discrepant, sequence results were used.

A variant was allocated to each episode of infection according to the following hierarchical process:

1. At least one sample within the identified episode of infection with confirmed wild-type or variant result: wild-type or variant assigned to the entire episode.
2. No samples within the identified episode of infection with confirmed wild-type or variant result, but the episode is within a household cluster with at least one known episode of infection with confirmed wild-type or variant result: cluster wild-type or variant assigned to the episode.
3. No samples within the identified episode of infection with confirmed wild-type or variant result, and the episode is not within a household cluster with at least one known episode of infection with confirmed wild-type or variant result: wild-type or variant assigned to the episode based on wave as a proxy for lineage circulation (i.e., wave 1: wild-type; wave 2: Beta variant; wave 3: Delta variant).

Cumulative incidence was estimated as the total number of individuals experiencing at least one episode divided by the total number of individuals.

For the analysis of factors associated with SARS-CoV-2 infection on PCR or serology we assessed overdispersion starting with a negative binomial model and retained a simpler Poisson model as there was no statistically significant overdispersion detected. For proportions 95% confidence intervals were estimated using the svy command in Stata, accounting for clustering by site and household.

Cluster duration was estimated as the interval from the first day of rRT-PCR positivity of the first individual in a cluster to the last day of rRT-PCR positivity of the last individual in that cluster. Using these definitions, it was possible for a household to experience >1 cluster of infections. Households with co-primary index cases (two individuals rRT-PCR positive for the first time on the same visit) or multiple SARS-CoV-2 variants in the same cluster were excluded from the analysis of household cumulative infection risk (HCIR). For analysis of factors associated with HCIR we excluded households with multiple SARS-CoV-2 variants in the same cluster. We excluded 5 clusters where more than one SARS-CoV-2 variant was identified in one cluster: 1) Agincourt household with 1 Beta, 3 Delta and 1

unassigned; 2) Agincourt household with 1 Beta and 1 Delta; 3) Klerksdorp household with 1 Beta and 1 Delta; 4) Klerksdorp household with 6 Alpha and 3 Beta; 5) Klerksdorp household with 1 Alpha, 4 Delta and one unassigned variant infections. We also excluded households that had co-primary index cases and those with no susceptible household member during the cluster period (eg rRT-PCR before the cluster.

The generation interval was calculated as the difference between the date of the first positive rRT-PCR in the index and the secondary infection within a household cluster. Following examination of the distribution of calculated generation intervals, we included all secondary infections with rRT-PCR positivity ≤21 days after the index case onset for analysis of factors associated with generation interval.

For multivariable models, we examined factors associated with several different outcomes, therefore the selected predictors varied across models. For analyses by age group, in each analysis we chose as reference the age group with the lowest prevalence of the outcome of interest.

Ethics

The U.S. Centers for Disease Control and Prevention's Institutional Review Board relied on the local review (#6840).

Disclaimer

The findings and conclusions in this report are those of the author(s) and do not necessarily represent the official position of the funding agencies or the Centers for Disease Control and Prevention.

Supplementary results

Adherence

The median swab adherence rate was 94% (interquartile range (IQR) 72%-97%) among individuals aged <19 years and 92% (IQR 56%-96%) among individuals aged ≥19 years.

Characteristics of reinfections

There were 87 reinfections identified during follow up (10 possible, 20 probable and 57 confirmed), 31 at the rural site and 56 at the urban site. Median age was 24 years (range 1-71 years), 58 (67%) were female, 16 (20%) of 81 with available data were PLHIV of whom 15 had data on CD4+ T cell count and HIV viral load and 1 had CD4+T cell count <200 cells/mm^3 and 4 had ≥1000 HIV viral copies per ml plasma. Eight (9%) individuals with reinfection had non-HIV underlying illness.

Symptoms and illness severity

Of 662 episodes, that occurred >14 days after the start of follow-up, 15% (97/662) of individuals reported ≥1 symptom, with 5% (30/662) reporting 1 symptom and 10% (67/662) reporting ≥2 symptoms. The most commonly reported symptoms were cough (12%, 78/662), runny nose (7%, 43/662) and headache (4%, 24/598). Among 662 episodes of infection, only 3% (17/662) reported difficulty breathing, 2% (14/662) reported fever and 2% (12/662) reported fever with cough. Other symptoms reported were diarrhoea 1% (4/662), vomiting 1% (5/662), abdominal pain 1% (4/598), sore throat 3% (18/598), body pains 2% (11/598), fatigue 3% (20/598), loss of smell or taste 2% (10/598) and confusion <1% (2/598).

There were 9 hospitalised individuals, all at the urban site. Median age of hospitalised individuals was 62.2 years (range 38-79 years) and 4 (4/9) were female. Of the nine hospitalised individuals one (1/7) was PLHIV, 4 (4/9) had non-HIV underlying illness, 4 (4/9) were overweight and 4 (4/9) were obese, none (0/7) had a smoking history and 5 (5/9) reported previous alcohol use.

There were two individuals who died, both at the urban site. The first was a 68 year old HIV-uninfected woman with underlying obesity (body mass index 42) and hypertension. She had symptoms of cough and difficulty breathing, she was not hospitalised and died 14 days after the infection episode onset. The second was a 75 year old HIV-uninfected man with no underlying illness. He had symptoms of fever, cough and difficulty breathing was hospitalised for severe hypoxia and admitted to the intensive care unit but did not receive mechanical ventilation. He died 7 days after the infection episode onset.

Characteristics of index cases vs other cases within clusters

When compared to non-index cases, index cases within household clusters were more likely to be aged 13-59 years compared to <5 years (Supplementary table 5).

Supplementary table 1: Symptom data collected among children aged <5 years and individuals aged ≥5 years in the PHIRST-C study

Symptom	Age group	
	**<5 years**	**≥5 years**
Measured fever	Collected	Collected
Reported fever	Collected	Collected
Cough	Collected	Collected
Shortness of breath	Not collected	Collected
Difficulty breathing or chest in drawing	Collected	Not collected
Sore throat	Not collected	Collected
Nasal congestion or runny nose	Collected	Collected
Lost sense of smell or taste	Not collected	Collected
Feeding poorly or had little appetite	Collected	Not collected
Vomiting	Collected	Collected
Diarrhoea (3 or more loose stools in 24 hours)	Collected	Collected
Abdominal pain	Not collected	Collected
Muscle aches	Not collected	Collected
Fatigue for one or more days	Not collected	Collected
Headache	Not collected	Collected
Been confused or unable to respond to questions	Not collected	Collected
Irritable or inconsolable	Collected	Not collected
Lethargic (unable to walk, sit, feed)	Collected	Not collected
Other symptoms (free text field)	Collected	Collected

Supplementary table 2: Baseline characteristics of households and individuals included in a Prospective Household cohort study of Influenza, Respiratory Syncytial virus and other respiratory pathogens community burden and Transmission dynamics in South Africa – COVID version (PHIRST-C) in a rural and an urban community, South Africa, 2020-2021

Characteristic	Overall n (%) or median (IQR)	Rural n (%) or median (IQR)	Urban n (%) or median (IQR)	P value
**Household level characteristics**	**N=222**	**N=114**	**N=108**	
Number of household members				
3-5	131 (59)	62 (54)	69 (60)	0.26
6-10	82 (37)	48 (42)	34 (31)	
>10	9 (4)	4 (4)	5 (5)	
Median number of household members	5 (4-7)	5 (4-7)	5 (4-6)	0.13
Number of rooms				
1-4	76 (34)	38 (33)	38 (35)	0.11
5-9	138 (62)	69 (61)	69 (64)	
≥10	8 (4)	7 (6)	1 (1)	
Median number of rooms	5 (4-7)	5 (3-8)	5 (4-6)	0.13
Number of rooms for sleeping				
1-2	93 (42)	37 (32)	56 (52)	<0.0001
3-4	106 (48)	57 (50)	49 (45)	
>4	23 (10)	20 (18)	3 (3)	
Median number of rooms for sleeping	3 (2-4)	3 (2-4)	2 (2-3)	<0.0001
Crowding (>2 people/sleeping room)	83 (37)	38 (33)	45 (42)	0.200
Child aged <5 years in house	109 (49)	67 (59)	42 (39)	0.003
Household member smokes indoors	59 (27)	13 (11)	46 (43)	<0.0001
Main water source tap inside (vs tap outside)	135 (61)	58 (51)	77 (71)	0.002
Handwashing place with water in house	207 (93)	103 (90)	104 (96)	0.078
Main fuel for cooking				

Electricity	141 (64)	35 (31)	106 (98)	<0.001
Wood	79 (36)	79 (69)	0 (0)	
Paraffin/gas/other	2 (1)	0 (0)	2 (2)	
Monthly household income[a]				
≤R800 (<USD54)	12 (6)	8 (7)	4 (4)	0.108
R801-R1600 (USD55-108)	38 (18)	15 (13)	23 (24)	
R1601-R3200 (USD109-116)	90 (43)	48 (42)	42 (45)	
R3201-R6400 (USD117-232)	54 (26)	33 (29)	21 (22)	
R6401-R12800 (USD233-464)	11 (5)	9 (8)	2 (2)	
>R12800 (>USD464)	3 (1)	1 (1)	2 (2)	
**Individual level characteristics**	**N=1200**	**N=643**	**N=557**	
Age group (years)				
<5	154 (13)	99 (15)	55 (10)	<0.0001
5-12	340 (28)	211 (33)	129 (23)	
13-18	170 (14)	88 (14)	82 (15)	
19-39	265 (22)	131 (20)	134 (24)	
40-59	168 (14)	68 (11)	100 (18)	
≥60	103 (9)	46 (7)	57 (10)	
Female sex	717 (60)	409 (64)	308 (55)	0.003
Level of education[b]				
No schooling	59 (11)	35 (14)	24 (8)	<0.001
Primary schooling	89 (17)	44 (18)	45 (16)	
Some secondary	230 (43)	83 (34)	147 (51)	
Secondary completed	142 (27)	80 (33)	62 (21)	
Post-secondary	15 (3)	3 (1)	12 (4)	
Employment[b]				
Unemployed	352 (66)	171 (70)	181 (62)	0.031
Employed	27 (5)	10 (4)	17 (6)	
Student	109 (20)	38 (16)	71 (24)	
Pensioner	47 (9)	26 (11)	21 (7)	
Reported alcohol use[c]	196 (30)	37 (12)	159 (47)	<0.0001
Reported current cigarette smoking[c]	124 (19)	12 (4)	112 (33)	<0.0001
HIV status[d]				
Uninfected	971 (85)	520 (86)	451 (83)	0.154

Infected	176 (15)	84 (14)	92 (17)	
Unknown	53	39	14	
HIV viral load[e]				
≥400 copies/ml	31 (19)	11 (14)	20 (21)	0.125
CD4+ T cell count[f]				
<200/ml	14 (8)	5 (6)	9 (11)	0.318
Previous tuberculosis	40 (3)	11 (2)	29(5)	0.001
Current tuberculosis	5 (<1)	1 (<1)	4 (1)	0.131
Other underlying illness[g]	125 (10)	42 (7)	83 (15)	<0.0001
Influenza vaccination 2020	22 (2)	18 (3)	4 (1)	0.008
Influenza vaccination 2021	7 (1)	6 (1)	1 (<1)	0.090
Fully vaccinated against SARS-CoV-2 vaccine by end of follow up[l]	57 (5)	23 (4)	34 (6)	0.040
Pneumococcal vaccine up to date for age[j]				
Yes	109 (92)	73 (97)	36 (84)	0.007
No	9 (8)	2 (3)	7 (16)	
No data	36	24	12	
DTaP-IPV/Hib vaccine up to date for age[i]				
Yes	113 (96)	75 (100)	38 (88)	0.003
No	5 (4)	0 (0)	5 (12)	
No data	36	24	12	

HIV – Human immunodeficiency virus, DTaP-IPV/Hib – Diphtheria, tetanus, acellular pertussis, inactivated polio, *Haemophilus influenzae* type B vaccine, IQR – interquartile range, OR – odds ratio, CI – confidence interval, NE – not estimated, n - number, USD – United States Dollar. Penalized logistic regression used for cells with zero values.

[a]Data available for 208 households, 114 rural and 94 urban [b]Individuals aged >18 years with available data N=535, 245 at rural site and 290 at urban site [c]Individuals aged ≥15 years N=643, 303 at rural site and 340 at urban site [d]% and p value among individuals with known status [e]Among 176 PLHIV, 166 (94%) reported currently receiving antiretroviral treatment (ART), of 165 PLHIV with data on CD4+ T cell count, 151 (92%) were >200 /ml, of 166 individuals with viral load data, 136 (82%) had <400 copies/ml [g]Self-reported history of asthma, lung disease, heart disease, stroke, spinal cord injury, epilepsy, organ transplant, immunosuppressive therapy, organ transplantation, cancer, liver

disease, renal disease or diabetes [j]Individuals aged <5 years N=154, 99 at rural site and 55 at urban site, 118 with available vaccination data, 75 at the rural site and 43 at the urban site [k]Estimated using logistic regression adjusted for clustering by site and household [l]Of 57 individuals who were fully vaccinated by the end of follow up, 23 received a single dose of the Johnson and Johnson vaccine and 34 received 2 doses of Pfizer vaccine and 47 (82%) were vaccinated from June through September. An additional 58 individuals received the first dose of Pfizer vaccine during the follow up period.

Supplementary Table 3: Factors associated with repeat infection with severe acute respiratory syndrome coronavirus 2 (SARS-CoV-2) infection on real-time reverse transcription polymerase chain reaction (rRT-PCR) and/or serology among 749 individuals with at least one infection in a rural and an urban community, South Africa, 2020-2021

Variable		SARS-CoV-2 reinfection n/N (%)	Univariate RR[e] (95% CI)	Multivariable aRR[e] (95% CI)
Site	Rural	32/368 (9)	Reference	Reference
	Urban	56/381 (15)	1.9 (1.1-3.0)	1.8 (1.1-3.0)
Age group (years)	<5	4/75 (5)	Reference	Reference
	5-12	18/205 (9)	1.6 (0.5-5.1)	1.7 (0.5-5.1)
	13-18	24/132 (18)	4.0 (1.3-12.5)	3.8 (1.3-11.8)
	19-39	25/165 (15)	3.0 (0.9-9.3)	2.9 (0.9-8.5)
	40-59	12/115 (10)	2.0 (0.6-6.7)	1.8 (0.5-5.9)
	≥60	5/57 ((9)	1.5 (0.4-6.3)	1.4 (0.4-5.7)
Sex	Female	58/454 (13)	1.4 (0.8-2.2)	
	Male	30/295 (10)	Reference	
HIV and viral load copies/ml[a]	Uninfected	66/608 (11)	Reference	
	Infected <400	11/87 (13)	1.2 (0.6-2.5)	
	Infected ≥400	4/22 (18)	1.9 (0.6-6.1)	
	HIV and/or viral load unknown	7/32 (22)	2.5 (0.9-6.4)	
HIV and CD4+ T cell count/ml[b]	Uninfected	66/608 (11)	Reference	
	Infected ≥200	14/99 (14)	1.4 (0.7-2.6)	
	Infected <200	1/8 (13)	1.3 (0.1-11.9)	
	HIV and/or CD4+ unknown	7/34 (21)	2.3 (0.9-5.7)	
Other underlying illness[c]	Absent	80/672 (12)	Reference	
	Present	8/77 (10)	0.8 (0.4-1.9)	
BMI[d]	Underweight	7/55 (13)	1.3 (0.5-3.2)	
	Normal weight	37/371 (10)	Reference	
	Overweight	19/150 (13)	1.2 (0.7-2.3)	
	Obese	24/171 (14)	1.4 (0.8-2.6)	

Number of individuals in household	3-5	39/311 (13)	Reference
	6-10	42/369 (11)	0.9 (0.5-1.5)
	≥11	7/69 (10)	0.7 (0.3-1.9)
Crowding (>2 people/sleeping room)	No	45/381 (12)	Reference
	Yes	43/368 (12)	1.0 (0.6-1.6)

HIV – Human immunodeficiency virus, BMI – Body mass index, RR – relative risk

Additional variables evaluated but not found to be significant on univariate or multivariable analysis: use of alcohol, current or previous smoking, current or previous tuberculosis, household income, fuel used for cooking, main water source, SARS-CoV-2 vaccination

[a]HIV data available for 719/749 (96%) of individuals. Among 111 PLHIV, 109 (98%) had available data on HIV viral load [b]Among 111 PLHIV, 107 (98%) had available data on CD4+ T cell count [c]Self-reported history of asthma, lung disease, heart disease, stroke, spinal cord injury, epilepsy, organ transplant, immunosuppressive therapy, organ transplantation, cancer, liver disease, renal disease or diabetes [d]BMI=body mass index calculated using the formula (weight in kilograms)/(height in metres squared). We defined BMI categories as follows: underweight - age <18 years weight for age or BMI <-2 standard deviations of the World Health Organization (WHO) Child Growth Standards, age ≥18 years BMI <18.5kg/m2; overweight - age <18 years BMI >+1 and ≤+2 standard deviations of the WHO growth standards, age ≥18 years BMI ≥25 and <30kg/m2, obese – age <18 years BMI >+2 standard deviations of the WHO growth standards, age ≥18 years BMI ≥30 kg/m2 [e]Estimated using logistic regression adjusted for clustering by site and household

Supplementary table 4: Characteristics associated with failure to develop a detectable serologic response for 294 individuals who had a negative serology result preceding a rRT-PCR confirmed infection with SARS-CoV-2 in a rural and an urban site, South Africa, 2020-2021[a]

Variable		No serologic response detected n/N (%)	Univariate RR (95% CI)	Multivariable aRR (95% CI)
Age group (years)	<5	6/30 (20)	4.9 (1.2-19.4)	18.8 (3.1-114.8)
	5-12	3/67 (4)	Reference	Reference
	13-18	3/49 (6)	1.4 (0.3-6.4)	3.7 (0.6-22.9)
	19-39	8/75 (11)	2.3 (0.6-8.6)	6.0 (1.2-29.0)
	40-59	5/45 (11)	2.5 (0.6-10.1)	7.0 (1.2-40.6)
	≥60	2/28 (7)	1.7 (0.3-9.4)	2.5 (0.3-19.1)
Sex	Female	20/187 (11)	1.6 (0.7-3.9)	
	Male	7/107 (7)	Reference	
HIV and viral load copies/ml[c]	Uninfected	23/237 (10)	Reference	
	Infected <400	2/33 (6)	0.7 (0.2-2.8)	
	Infected ≥400	2/16 (13)	1.6 (0.4-6.4)	
	HIV and/or viral load unknown	0/8 (0)	Not estimated	
HIV and CD4+ T cell count/ml[c]	Uninfected	23/237 (10)	Reference	
	Infected ≥200	3/43 (7)	0.8 (0.2-2.5)	
	Infected <200	1/4 (25)	3.9 (0.5-27.6)	
	HIV and/or CD4+ unknown	0/10 (0)	Not estimated	
Other underlying illness[b]	No	23/262 (9)	Reference	
	Yes	4/32 (13)	1.6 (0.5-4.7)	
Symptoms	Absent	24/245 (10)	Reference	
	Present	3/49 (6)	0.7 (0.2-2.3)	
Duration of viral RNA shedding (days)	≤4	21/49 (43)	Reference	Reference
	>4	6/245 (2)	0.1 (0.1-0.1)	0.1 (0.0-0.2)

Minimum Ct	≤30	10/253 (4)	0.1 (0.0-0.1)	0.2 (0.1-0.1)
value	>30	17/41 (41)	Reference	Reference

[a]Estimated using logistic regression adjusted for clustering by site and household [b]Self-reported history of asthma, lung disease, heart disease, stroke, spinal cord injury, epilepsy, organ transplant, immunosuppressive therapy, organ transplantation, cancer, liver disease, renal disease or diabetes [c]HIV data available for 286 (97%) of 294 included individuals, of 49 HIV-infected individuals viral load data available for 49 (100%) and CD4+ T cell count data available for 47 (96%)

Supplementary table4: Factors associated with generation interval among 198 index household contact pairs with interval ≤21 days at a rural and an urban site, South Africa, 2020-2021[a]

Variable		Interval (days) Mean±SD (Range)	Univariate HR	Multivariable aHR
**Characteristics of the index case**				
Age group (years)	<5	7.0±4.8 (2-18)	0.7 (0.1-1.7)	
	5-12	6.6±4.2 (2-20)	0.8 (0.4-1.8)	
	13-18	9.3±5.7 (2-21)	0.5 (0.2-0.9)	
	19-39	6.6±4.0 (2-19)	0.9 (0.4-1.8)	
	40-59	7.7±4.4 (2-20)	0.7 (0.3-1.4)	
	≥60	6.5±2.4 (3-10)	Reference	
Sex	Female	7.1±4.4 (2-21)	1.4 (1.0-1.8)	
	Male	8.5±5.1 (2-21)	Reference	
HIV and viral load copies/ml	Uninfected	7.6±4.9 (2-21)	Reference	
	Infected <400	7.4±4.2 (3-16)	1.1 (0.8-1.6)	
	Infected ≥400	7.0±5.0 (2-12)	1.2 (0.4-3.7)	
	HIV and/or viral load unknown	6.1±2.3 (3-10)	1.7 (0.8-3.7)	
HIV and CD4+ T cell count/ml	Uninfected	7.6±4.9 (2-21)	Reference	
	Infected ≥200	6.7±3.8 (2-15)	1.3 (0.9-1.9)	
	Infected <200	13.7±2.1 (12-16)	0.5 (0.1-1.4)	
	HIV and/or CD4+ unknown	6.3±2.1 (3-10)	1.7 (0.8-3.5)	
Symptoms	Absent	7.7±4.8 (2-21)	Reference	Reference
	Present	5.6±3.6 (3-15)	1.7 (1.1-2.7)	1.7 (1.1-2.7)
Minimum Ct value	≤30	7.6±4.8 (2-21)	8 (0.5-1.4)	
	>30	7.0±3.1 (3-14)	Reference	
Duration of shedding (days)	≤4	5.4±3.4 (2-14)	Reference	Reference
	>4	7.7±4.8 (2-21)	0.5 (0.3-0.9)	0.6 (0.3-0.9)
Epidemic wave	1	4.8±2.1 (2-8)	Reference	
	2	8.2±4.8 (2-21)	0.4 (0.2-0.6)	
	3	7.4±4.8 (2-21)	0.4 (0.2-0.7)	
Variant	Wild type	4.7±2.2 (2-8)	Reference	Reference
	Beta	7.8±4.5 (2-21)	0.4 (0.2-0.7)	0.2 (0.1-0.6)

	Alpha	12.8±4.4 (8-17)	0.2 (0.1-0.6)	0.4 (0.2-0.6)
	Delta	7.5±4.9 (2-21)	0.4 (0.2-0.7)	0.4 (0.2-0.7)
**Characteristics of the household member**				
Age group (years)	<5	7.9±5.1 (2-21)	0.8 (0.5-1.3)	
	5-12	7.0±4.4 (2-21)	Reference	
	13-18	7.9±5.3 (2-21)	0.8 (0.5-1.2)	
	19-39	7.1±4.6 (3-19)	1.0 (0.6-1.5)	
	40-59	8.1±4.7 (2-20)	0.8 (0.5-1.3)	
	≥60	8.4±4.7 (2-17)	0.8 (0.4-1.4)	
Sex	Female	7.2±4.6 (2-21)	1.2 (0.9-1.5)	
	Male	8.0±4.8 (2-21)	Reference	
HIV and viral load copies/ml	Uninfected	7.5±4.8 (2-21)	Reference	
	Infected <400	7.3±4.1 (3-17)	1.1 (0.7-1.9)	
	Infected ≥400	8.9±5.6 (3-17)	0.8 (0.4-1.6)	
	HIV and/or viral load unknown		1.4 (0.7-2.9)	
HIV and CD4+ T cell count/ml	Uninfected	7.5±4.8 (2-21)	Reference	
	Infected ≥200	7.5±4.5 (3-17)	0.8 (0.6-1.3)	
	Infected <200	14±0.0 (14-14)	0.4 (0.1-3.0)	
	HIV and/or CD4+ unknown	6.3±4.1 (3-12)	1.4 (0.6-3.0)	

SD – Standard deviation [a]Estimated using Weibull accelerated failure time regression adjusted for clustering by site and household. Samples were collected at 2 to 4 day intervals. Serial interval refers to the interval between first positive influenza result in the index case and the secondary case. Additional factors evaluated but not found to be statistically significant include year, site, employment of index or contact, education level of index or contact, alcohol or smoking of index or contact, other underlying illness of index or contact, body mass index of index or contact, receipt of influenza vaccine of index or contact, number of people in household, number of rooms, crowding, smoking inside the house.

Supplementary table 5: Characteristics associated with being an index case (vs non index case) for 171 household clusters of SARS-CoV-2 infection in a rural and an urban site, South Africa, 2020-2021[a]

Variable		Index case n/N (%)	Univariate RR (95% CI)	Multivariable aRR (95% CI)
Site	Rural	200/350 (57)	Reference	
	Urban	191/326 (59)	1.1 (0.8-1.4)	
Age group (years)	<5	27/67 (40)	Reference	Reference
	5-12	96/189 (51)	1.5 (0.9-2.7)	1.5 (0.8-2.7)
	13-18	79/127 (62)	2.4 (1.3-4.5)	2.4 (1.3-4.5)
	19-39	103/149 (69)	3.3 (1.8-6.0)	3.1 (1.6-5.9)
	40-59	56/89 (63)	2.5 (1.3-4.8)	2.2 (1.1-4.2)
	≥60	30/55 (55)	1.8 (0.9-3.7)	1.6 (0.7-3.4)
Sex	Female	231/412 (56)	0.8 (0.6-1.1)	
	Male	160/264 (61)	Reference	
HIV and viral load copies/ml	Uninfected	308/549 (56)	Reference	Reference
	Infected <400	49/71 (69)	1.7 (1.0-3.0)	1.4 (0.8-2.5)
	Infected ≥400	13/23 (57)	1.0 (0.4-2.4)	0.8 (0.3-2.0)
	HIV and/or viral load unknown	21/33 (64)	1.4 (0.7-2.9)	1.8 (0.9-3.6)
HIV and CD4+ T cell count/ml	Uninfected	308/549 (56)	Reference	
	Infected ≥200	54/85 (64)	1.4 (0.8-2.2)	
	Infected <200	6/7 (86)	4.7 (0.6-39.3)	
	HIV and/or CD4+ unknown	23/35 (66)	1.5 (0.7-3.1)	
Other underlying illness[b]	No	355/614 (58)	Reference	
	Yes	36/62 (58)	1.0 (0.6-1.7)	
BMI[c]	Underweight	26/53 (49)	0.8 (0.4-1.4)	1.6 (0.9-2.9)
	Normal weight	195/350 (56)	Reference	Reference
	Overweight	76/125 (61)	1.2 (0.8-1.9)	1.5 (0.7-2.9)
	Obese	93/145 (64)	1.4 (1.0-2.1)	2.0 (0.9-3.9)

[a]Estimated using logistic regression adjusted for clustering by site and household [b]Self-reported history of asthma, lung disease, heart disease, stroke, spinal cord injury, epilepsy, organ transplant, immunosuppressive therapy, organ transplantation, cancer, liver disease, renal disease or diabetes [c]BMI=body mass index calculated using the formula (weight in kilograms)/(height in metres squared). We defined BMI categories as follows: underweight - age <18 years weight for age or BMI <-2 standard deviations of the World Health Organization (WHO) Child Growth Standards, age ≥18 years BMI <18.5kg/m2; overweight - age <18 years BMI >+1 and ≤+2 standard deviations of the WHO growth standards, age ≥18 years BMI ≥25 and <30kg/m2, obese – age <18 years BMI >+2 standard deviations of the WHO growth standards, age ≥18 years BMI ≥30 kg/m2

Supplementary figure 1: Location of rural (Agincourt) and urban (Jouberton) study sites in South Africa

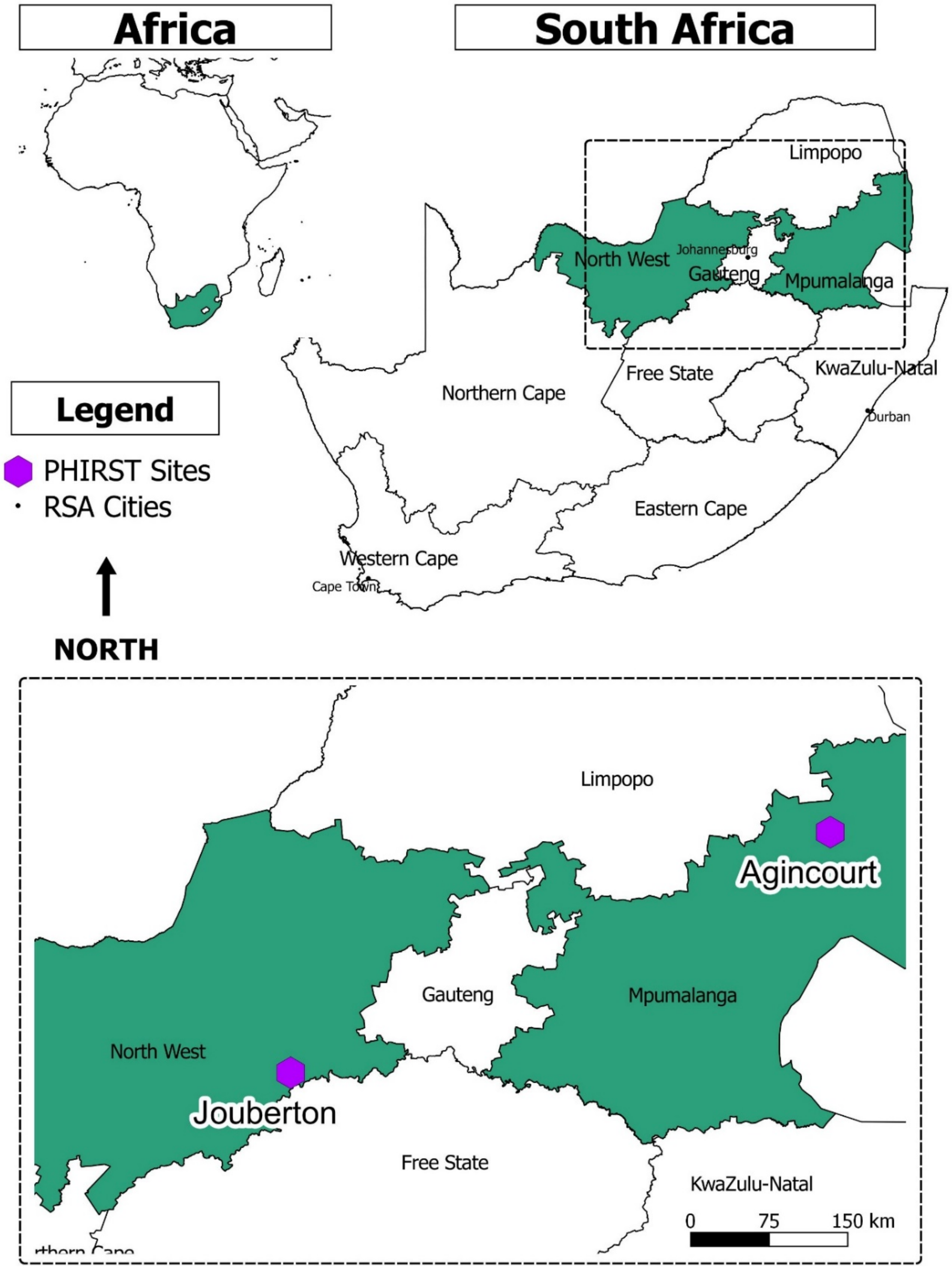

Supplementary figure 2: Methods for assigning episodes and clusters of infection.

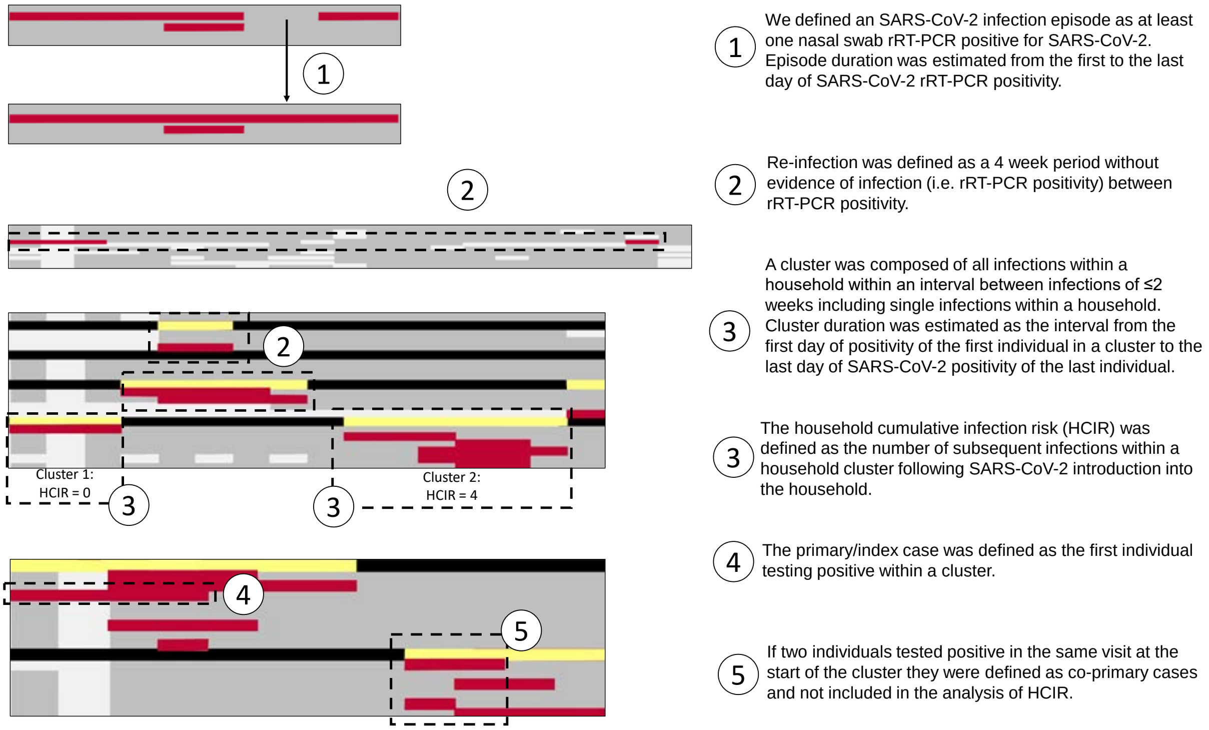

Figure legend

Red bar - Positive rRT-PCR

Dark Grey bar - Negative rRT-PCR

Light Grey bar - Missing swab

Black bar - No Household Cluster

Yellow bar - Household Cluster

Supplementary figure 3: Flow chart of individuals included in the study, a rural site and an urban site, South Africa, 2020-2021

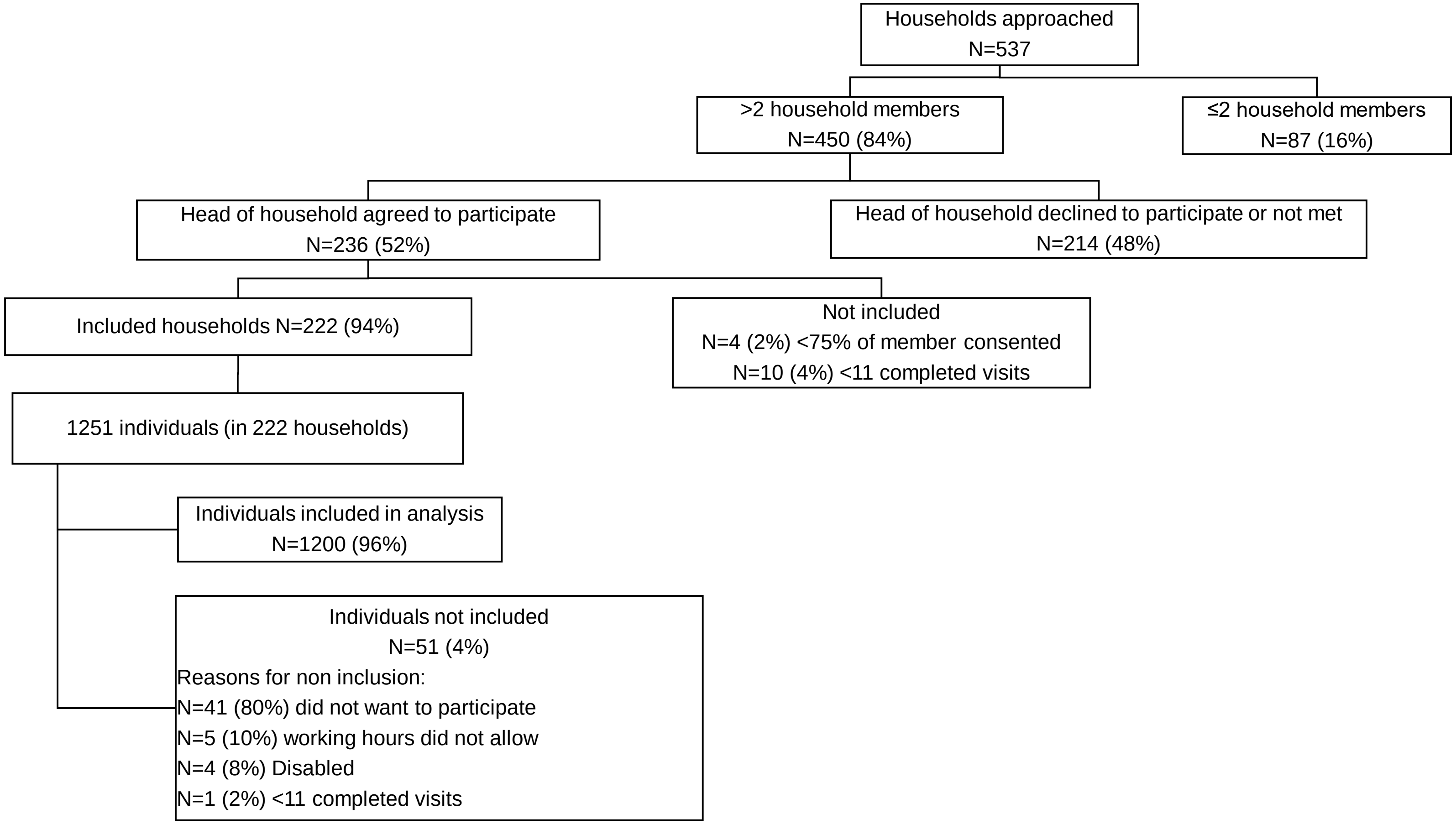

Supplementary figure 4: Proportion of individuals infected with SARS-CoV-2 at the end of follow up by method of diagnosis, age group and site at a rural and an urban site, South Africa, 2020-2021

a) Rural site b) Urban site

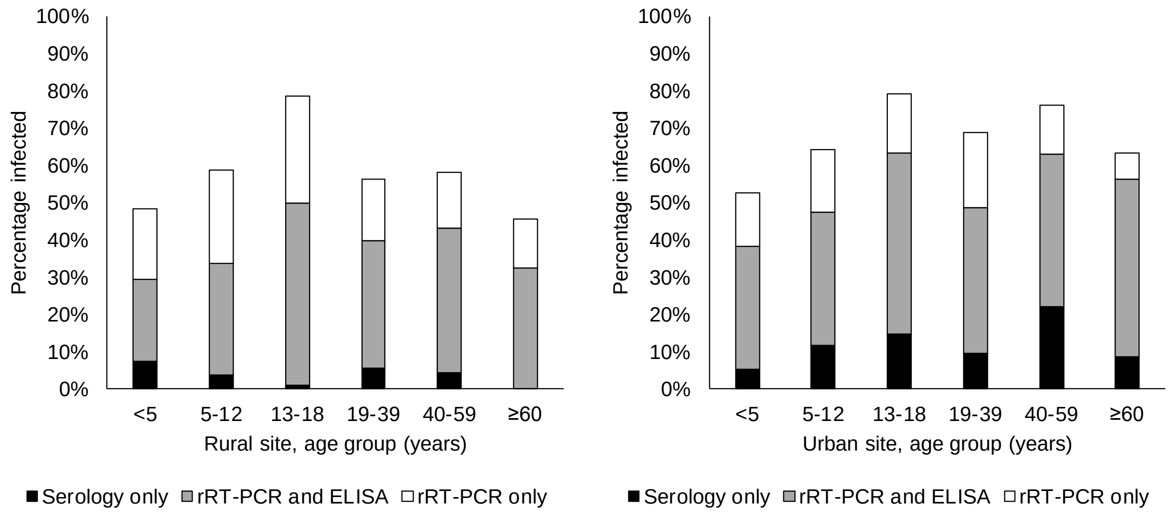

rRT-PCR- real time reverse transcription polymerase chain reaction

Supplementary figure 5: SARS-CoV-2 episodes of infection by SARS-CoV-2 variant at the rural site (a) and urban site (b) in 2020-2021.

Columns are individual follow up visits and rows are individual participants. The white, light grey and coloured horizontal lines each denote an individual with in a household. Each column indicates an individual follow up visit. Follow up visits are coloured white if no sample was tested, light grey if the sample tested negative for SARS-CoV-2 and coloured if the nasopharyngeal swab tested positive for SARS-CoV-2. Colours are green for non variant, purple for Beta variant, orange for Alpha variant and yellow for Delta variant after imputation for missing data. Individuals in the same household make up sequential rows. A high resolution version of this figure has been provided separately.

A Rural site

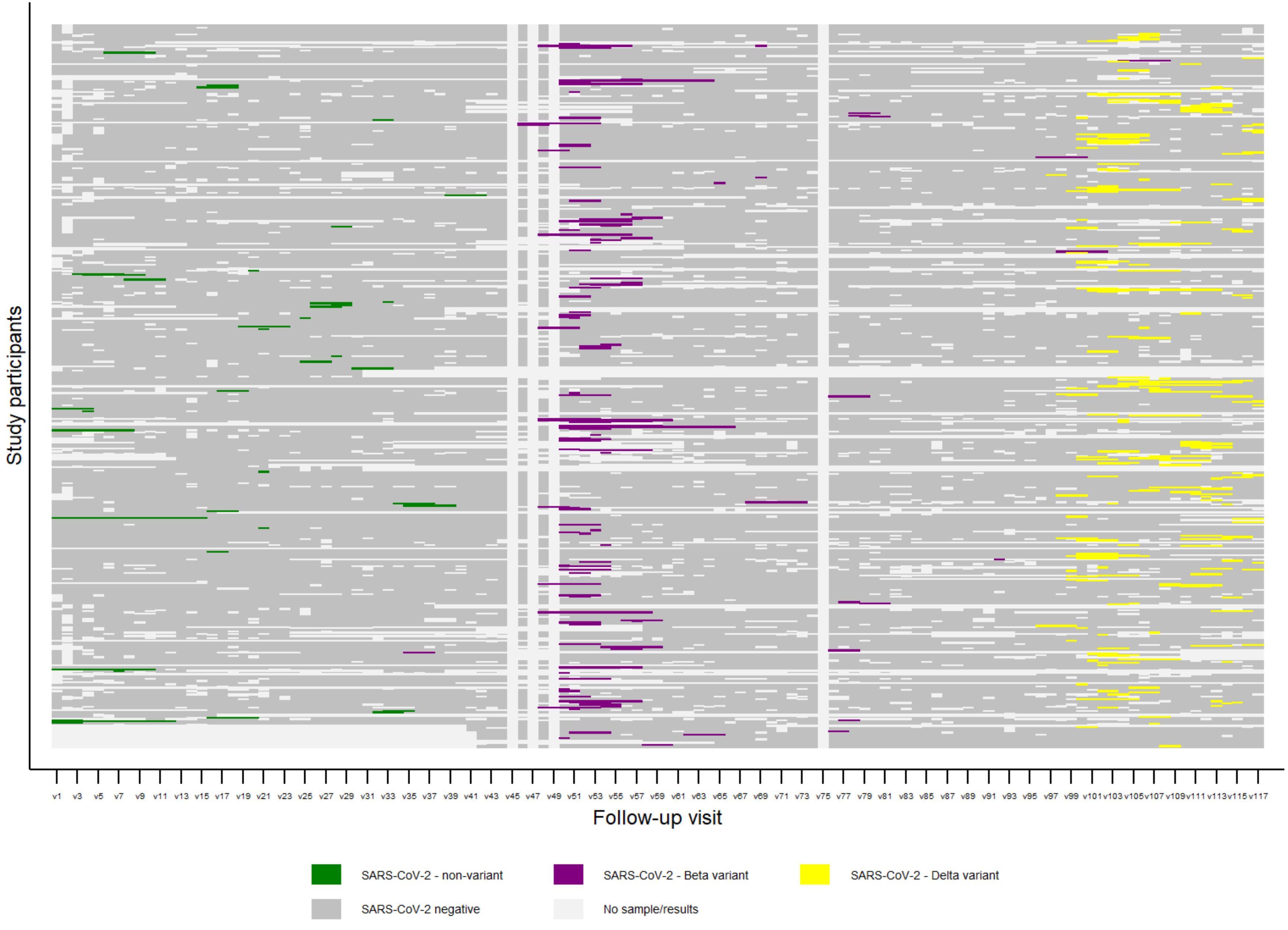
Study participants
v1 v3 v5 v7 v9 v11 v13 v15 v17 v19 v21 v23 v25 v27 v29 v31 v33 v35 v37 v39 v41 v43 v45 v47 v49 v51 v53 v55 v57 v59 v61 v63 v65 v67 v69 v71 v73 v75 v77 v79 v81 v83 v85 v87 v89 v91 v93 v95 v97 v99 v101 v103 v105 v107 v109 v111 v113 v115 v117
Follow-up visit
SARS-CoV-2 - non-variant
SARS-CoV-2 - Beta variant
SARS-CoV-2 - Delta variant
SARS-CoV-2 negative
No sample/results

B) Urban site

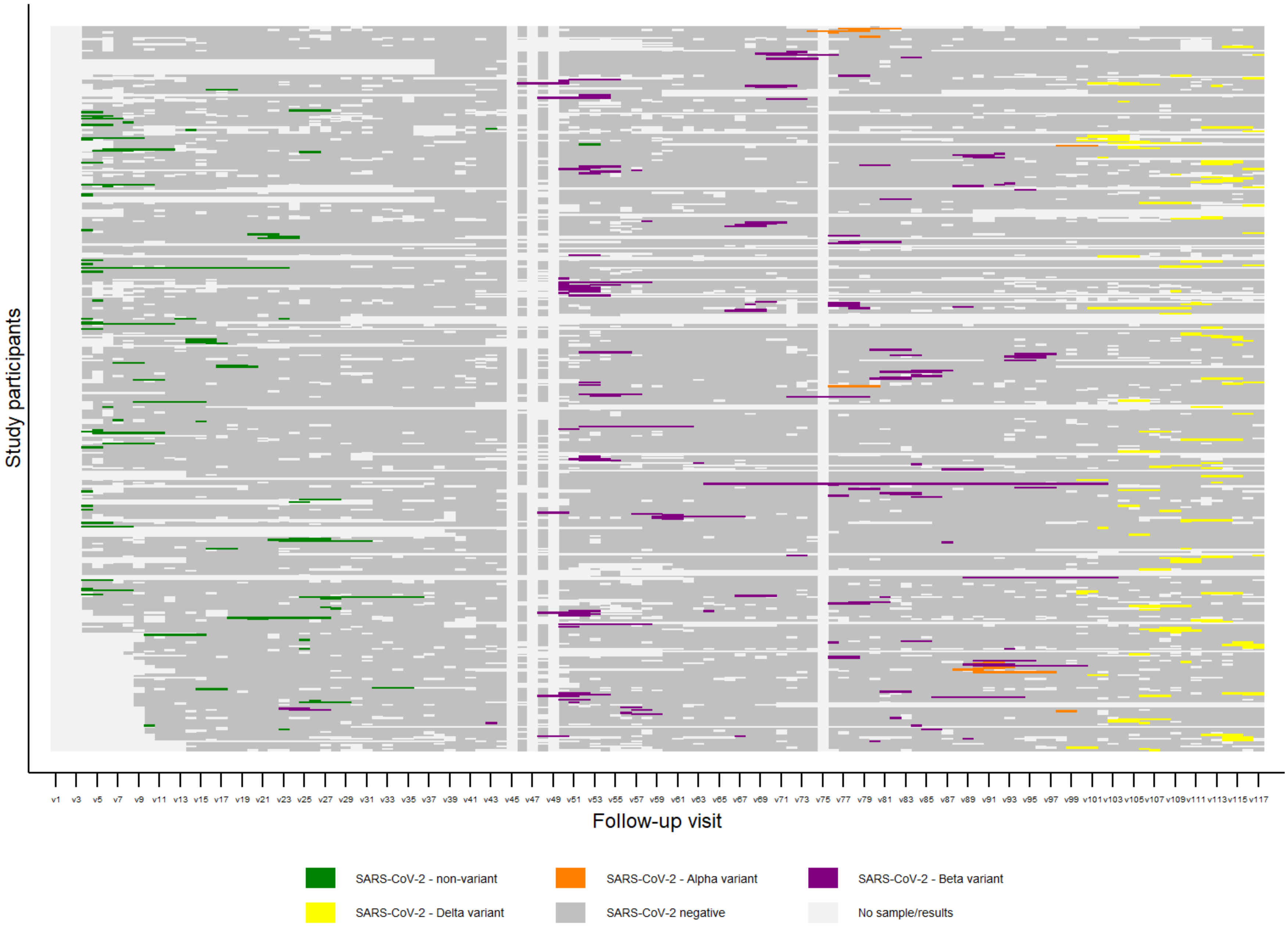
Study participants
Follow-up visit
v1 v3 v5 v7 v9 v11 v13 v15 v17 v19 v21 v23 v25 v27 v29 v31 v33 v35 v37 v39 v41 v43 v45 v47 v49 v51 v53 v55 v57 v59 v61 v63 v65 v67 v69 v71 v73 v75 v77 v79 v81 v83 v85 v87 v89 v91 v93 v95 v97 v99 v101 v103 v105 v107 v109 v111 v113 v115 v117
SARS-CoV-2 - non-variant
SARS-CoV-2 - Alpha variant
SARS-CoV-2 - Beta variant
SARS-CoV-2 - Delta variant
SARS-CoV-2 negative
No sample/results

Supplementary figure 6: Epidemic curve of rRT-PCR-confirmed SARS-CoV-2 by non-variant or variant type at a rural and an urban site, South Africa, 2020-2021*

A) Rural site

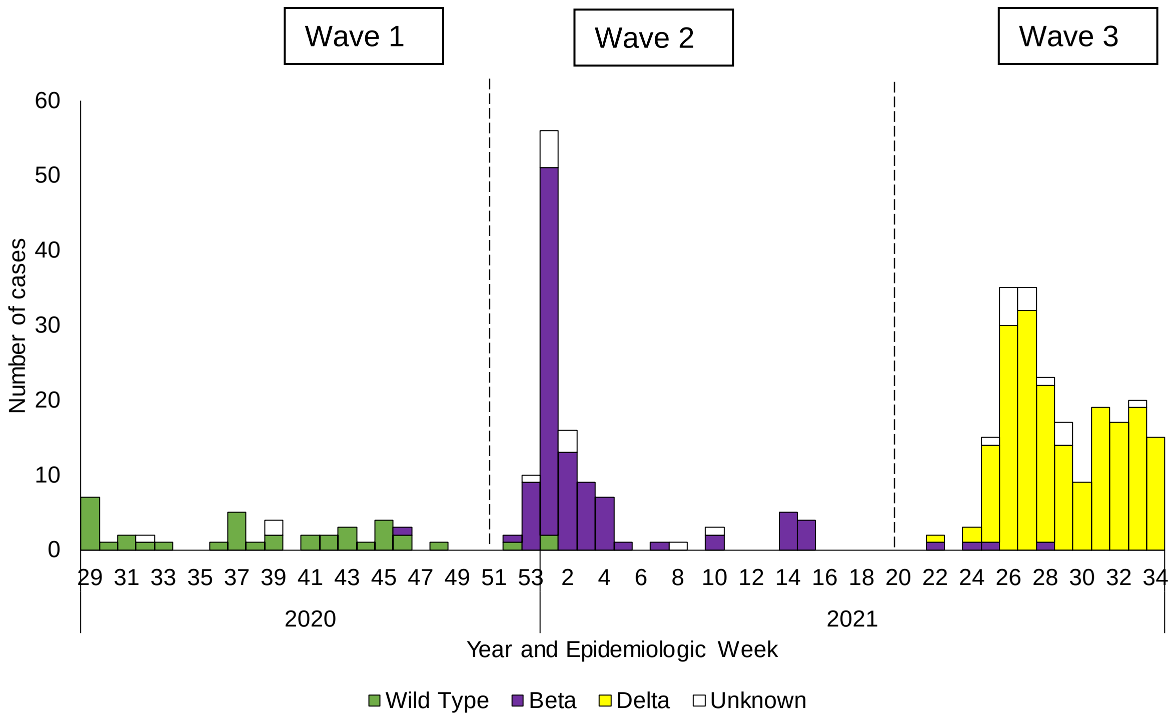

b) Urban site

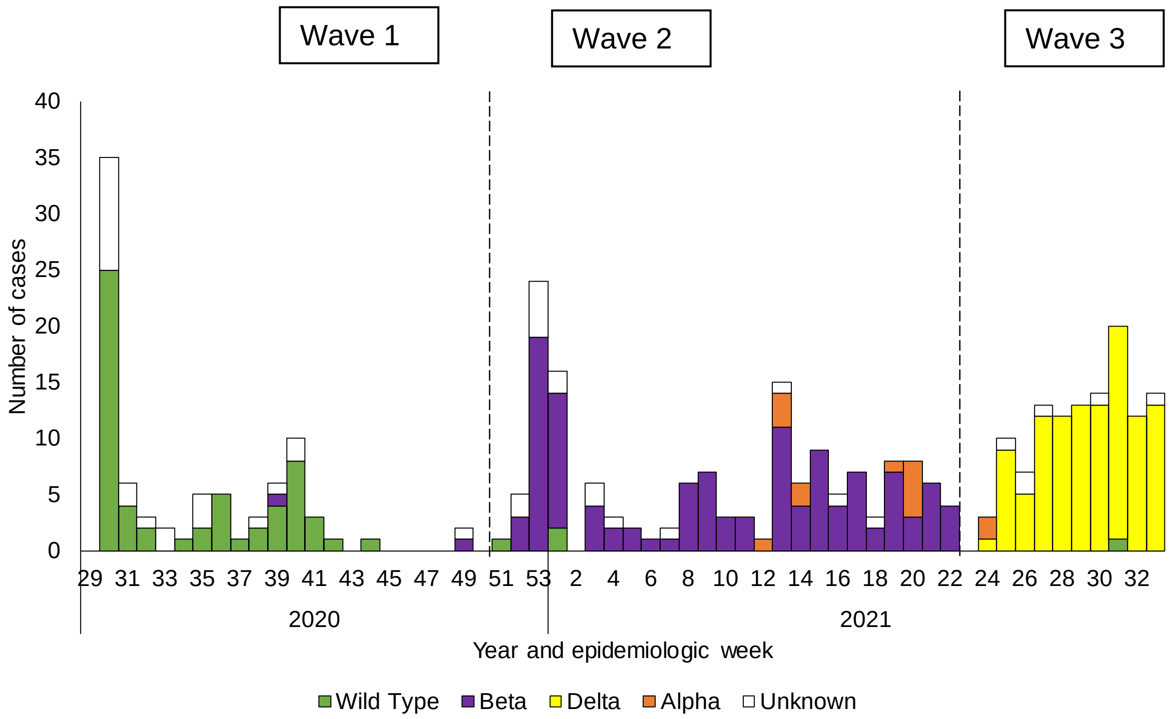

*Follow up began on 16 and 27 July 2020 at rural and urban site, respectively, vertical dashed lines indicates analysis cut off between first, second and third SARS-CoV-2 waves at each site

Supplementary figure 7: Interval between first SARS-CoV-2-positive real-time reverse transcription polymerase chain reaction (rRT-PCR) in the index case and first positive rRT-PCR in household contacts (generation interval), in a rural and an urban community, South Africa, 2020-2021 (n=212 case pairs, 198 included for generation interval estimation)

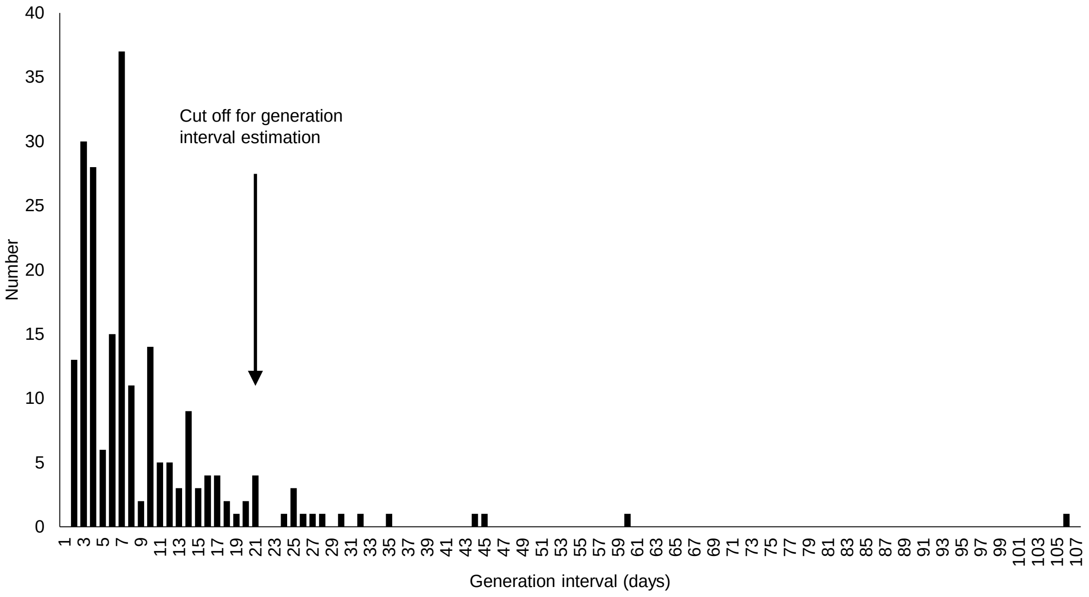

The PHIRST-C Group

Name	Affiliation
Jinal N. Bhiman	Centre for Respiratory Diseases and Meningitis, National Institute for Communicable Diseases of the National Health Laboratory Service, Johannesburg, South Africa. School of Pathology, Faculty of Health Sciences, University of the Witwatersrand, Johannesburg, South Africa.
Amelia Buys	Centre for Respiratory Diseases and Meningitis, National Institute for Communicable Diseases of the National Health Laboratory Service, Johannesburg, South Africa.
Maimuna Carrim	Centre for Respiratory Diseases and Meningitis, National Institute for Communicable Diseases of the National Health Laboratory Service, Johannesburg, South Africa. School of Pathology, Faculty of Health Sciences, University of the Witwatersrand, Johannesburg, South Africa.
Cheryl Cohen	Centre for Respiratory Diseases and Meningitis, National Institute for Communicable Diseases of the National Health Laboratory Service, Johannesburg, South Africa. School of Public Health, Faculty of Health Sciences, University of the Witwatersrand, Johannesburg, South Africa.
Linda de Gouveia	Centre for Respiratory Diseases and Meningitis, National Institute for Communicable Diseases of the National Health Laboratory Service, Johannesburg, South Africa.
Mignon du Plessis	Centre for Respiratory Diseases and Meningitis, National Institute for Communicable Diseases of the National Health Laboratory Service, Johannesburg, South Africa. School of Pathology, Faculty of Health Sciences, University of the Witwatersrand, Johannesburg, South Africa.
Jacques du Toit	MRC/Wits Rural Public Health and Health Transitions Research Unit (Agincourt), Faculty of Health Sciences, School of Public Health, University of the Witwatersrand, Johannesburg, South Africa.
Francesc Xavier Gómez-Olivé	MRC/Wits Rural Public Health and Health Transitions Research Unit (Agincourt), Faculty of Health Sciences, School of Public Health, University of the Witwatersrand, Johannesburg, South Africa.
Kathleen Kahn	MRC/Wits Rural Public Health and Health Transitions Research Unit (Agincourt), Faculty of Health Sciences, School of Public Health, University of the Witwatersrand, Johannesburg, South Africa.
Kgaugelo Patricia Kgasago	Perinatal HIV Research Unit (PHRU), University of the Witwatersrand, Johannesburg, South Africa.
Jackie Kleynhans	Centre for Respiratory Diseases and Meningitis, National Institute for Communicable Diseases of the National Health Laboratory Service, Johannesburg, South Africa. School of Public Health, Faculty of Health Sciences, University of the Witwatersrand, Johannesburg, South Africa.
Retshidisitswe Kotane	Centre for Respiratory Diseases and Meningitis, National Institute for Communicable Diseases of the National Health Laboratory Service, Johannesburg, South Africa.
Limakatso Lebina	Perinatal HIV Research Unit (PHRU), University of the Witwatersrand, Johannesburg, South Africa. Africa Health Research Institute, Durban, South Africa. Africa Health Research Institute, KwaZulu-Natal, South Africa.
Neil A. Martinson	Perinatal HIV Research Unit (PHRU), University of the Witwatersrand, Johannesburg, South Africa. Johns Hopkins University Center for TB Research, Baltimore, Maryland, United States of America.
Meredith L. McMorrow	Influenza Division, Centers for Disease Control and Prevention, Atlanta, Georgia, United States of America. Influenza Program, Centers for Disease Control and Prevention, Pretoria, South Africa.
Tumelo Moloantoa	Perinatal HIV Research Unit (PHRU), University of the Witwatersrand, Johannesburg, South Africa.
Jocelyn Moyes	Centre for Respiratory Diseases and Meningitis, National Institute for Communicable Diseases of the National Health Laboratory Service, Johannesburg, South Africa. School of Public Health, Faculty of Health Sciences, University of the Witwatersrand, Johannesburg, South Africa.
Stefano Tempia	Influenza Division, Centers for Disease Control and Prevention, Atlanta, Georgia, United States of America. Influenza Program, Centers for Disease Control and Prevention, Pretoria, South Africa. School of Public Health, Faculty of Health Sciences, University of the Witwatersrand, Johannesburg, South Africa.
Stephen Tollman	MRC/Wits Rural Public Health and Health Transitions Research Unit (Agincourt), School of Public Health, Faculty of Health Science, University of the Witwatersrand.
Anne von Gottberg	Centre for Respiratory Diseases and Meningitis, National Institute for Communicable Diseases of the National Health Laboratory Service, Johannesburg, South Africa. School of Pathology, Faculty of Health Sciences, University of the Witwatersrand, Johannesburg, South Africa.
Floidy Wafawanaka	MRC/Wits Rural Public Health and Health Transitions Research Unit (Agincourt), Faculty of Health Sciences, School of Public Health, University of the Witwatersrand, Johannesburg, South Africa.
Nicole Wolter	Centre for Respiratory Diseases and Meningitis, National Institute for Communicable Diseases of the National Health Laboratory Service, Johannesburg, South Africa. School of Pathology, Faculty of Health Sciences, University of the Witwatersrand, Johannesburg, South Africa.
